# NR2F2 in cancer-associated fibroblasts drives immune microenvironment remodeling and promotes lung adenocarcinoma progression

**DOI:** 10.3389/fimmu.2026.1776008

**Published:** 2026-04-13

**Authors:** Song Zhou, Yibo Guo, Runjie Cheng, Qianqian Zhang, Yuanxin Xing, Yizhen Geng, Jinze Yang, Xiaoguang Han, Ying Zhang, Wei Xie

**Affiliations:** 1 Central Hospital Affiliated to Shandong First Medical University, Shandong First Medical University & Shandong Academy of Medical Sciences, Jinan, Shandong, China; 2 Shandong University of Traditional Chinese Medicine, Jinan, Shandong, China; 3 Department of Spine Surgery, Beijing Jishuitan Hospital, Capital Medical University, Beijing, China; 4 The Second Affiliated Hospital of Shandong University of Traditional Chinese Medicine, Jinan, Shandong, China

**Keywords:** cancer-associated fibroblast, lung adenocarcinoma, molecular biomarkers, multi-omics analysis, Nr2f2, prognostic model, single-cell RNA sequencing, tumor microenvironment

## Abstract

**Background:**

The high heterogeneity of lung adenocarcinoma (LUAD) is largely due to its complex tumor immune microenvironment (TIME). Cancer-associated fibroblasts (CAFs) are a core matrix component of TIME. However, their functional heterogeneity and the specific molecular mechanisms driving tumor progression have not been fully elucidated. In addition, the role of nuclear receptor NR2F2 in tumor development is still controversial.

**Method:**

This study integrated scRNA-seq data from the GEO database with RNA-seq data from TCGA and GEO and then performed multiple levels of validation through *in vitro* experiments. We adopted a systematic computational biology strategy and analyzed the cellular composition, interaction networks and functional states of cancer-associated fibroblasts (CAFs) in lung adenocarcinoma using Seurat, CellChat, and AUCell. According to the marker genes of key CAF subgroup, prognostic risk models were constructed through LASSO-Cox regression and validated in an independent cohort (GSE72094). Afterwards, we carried out *in vitro* experiments and validated the biological role of NR2F2 through a coculture system. Functional validation was conducted through siRNA knockdown, plasmid overexpression, CCK-8 assay, EdU labeling, and Transwell experiments.

**Result:**

We noticed the CAF - 2 subgroup, characterized by the highest level of TGF - β signaling activation, sends various signals to different cell types. We constructed and verified a consistent prognostic signature made of 16 genes using the LASSO-Cox method. This model can effectively assess the risk of LUAD patients. The prognosis in high-risk group is worse. And we also do some analysis to find out that risk score is highly associated with immunosuppressive TME and high expressions of PD - L1. We have found in our further study that the expression of NR2F2 in CAF is associated with the promoting of matrix remodeling and metabolic reprogramming. From the coculture system and *in vitro* functional experiments, overexpression of NR2F2 in CAFs enhanced tumor cell proliferation and invasion, whereas knockdown of NR2F2 attenuated these malignant phenotypes.

**Conclusion:**

Using single-cell RNA sequencing data, we identified a CAF subgroup with the most active TGF-β signaling. Based on the marker genes of the subgroup, we constructed and validated an effective prognostic model, then we further screened and confirmed NR2F2 as a major pro-tumorigenic regulator from this feature gene set through single cell and transcriptome data as well as *in vitro* experiments. NR2F2 promotes malignant remodeling of TIME by synergistically enhancing TGF-β signaling and EMT processes. Our study provides not only a solid theoretical foundation but also a therapeutic target to explore new therapeutic options targeting the CAFs-TGF-β-EMT axis.

## 1 Introduction

Lung cancer is one of the top causes of cancer-related deaths in the world, and among different histological types of lung cancers, lung adenocarcinoma (LUAD) is most common (1, 2). Despite significant advancements in early detection and targeted therapy, the long-term survival of LUAD patients is still poor, which is mainly attributed to the high heterogeneity of the tumor as well as the complicated TIME (3).

TIME is a dynamic ecosystem. Within it, cancer-associated fibroblasts (CAFs) are the most abundant matrix component in TIME, playing a crucial role in tumor progression (4). In the past few years, scRNA-seq has revealed that CAFs are not a single population, but multiple functional subgroups with significant transcriptional heterogeneity (5, 6). However, the specific role of how these cells form immunosuppressive TME and affect the clinical prognosis remains a hot and challenging topic in current research.

Epithelial-mesenchymal transition (EMT) is a core mechanism that CAFs drive tumor progression (7). The characteristic of EMT is the loss of cell-cell adhesion, the acquisition of migratory and invasive properties (8). Notably, CAFs secrete a number of factors, such as HGF, TGF-β, to promote EMT in cancer cells. Moreover, CAFs remodel the TIME into a pro-tumorigenic environment (9). The core driving force for initiating and maintaining the EMT program is TGF-β signaling (10). As a major source of TGF-β, CAFs are considered to play a key role in remodeling the TIME, thereby promoting EMT and invasive ability in tumor cells. This mechanism has been supported in many cancers such as breast cancer (11), colorectal cancer (12) and pancreatic cancer (13). However, the upstream regulatory machinery within CAFs that controls and amplifies TGF-β signaling to drive EMT remains poorly understood.

NR2F2, also known as COUP-TFII, is a transcription factor that plays a critical role in embryonic development, cell differentiation, and metabolism (14). In cancer biology, it has been reported that NR2F2 has dual functions of inhibiting or promoting cancer, and the contradiction of this function may depend on the cancer types and cellular environment. The specific role and regulatory mechanism of NR2F2 in LUAD remain poorly defined.

To address these issues, this study integrated single-cell RNA sequencing data, bulk transcriptomics data, and machine learning approaches. We first employed scRNA-seq to analyze the environment of lung adenocarcinoma (LUAD), with a particular focus on the cancer-associated fibroblasts (CAFs). Through functional analysis and Cellchat analysis, we identified a subgroup of CAFs (CAF-2), which is characterized by a highly active TGF-β pathway. Subsequently, we translated this biological insight into a robust clinical prognostic model and systematically evaluated its association with tumor microenvironment characteristics and immunotherapy response. Finally, through further validation and *in vitro* experiments, we identified the regulatory effect of the core gene NR2F2 selected in the model on CAF biology and tumor cells. Starting from the characteristics of CAFs in LUAD, this study aims to develop a new prognostic tool and search potential therapeutic targets.

## 2 Methods

### 2.1 Data downloading and preprocessing

Nine lung adenocarcinoma samples and two normal samples were screened from the GEO database (datasets: GSE189357, GSE149655; Platform: GPL24676 Illumina NovaSeq 6000) for single-cell transcriptome analysis (15, 16). The bulk RNA-seq data and corresponding clinical information for lung adenocarcinoma were obtained from the TCGA database (17). To verify the accuracy of the prognostic model, we used the GSE72094 dataset (18) (platform: GPL15048); The expression profile and clinical information of 442 lung adenocarcinoma patients were included as an external validation set.

### 2.2 Single cell RNA sequencing data processing and cell annotation

We used R software (version 4.5.1) and Seurat package (version 5.1.0) (19) to process and analyze single-cell transcriptome data. After rigorous quality control, cells expressing at least three genes were retained. Besides, the number of genes detected per cell ranges from 500 to 6000. Cells with a mitochondrial gene ratio less than 20% were preserved. We then used the Normalize Data function to eliminate sequencing depth and batch effects. Principal component analysis (PCA) was performed for dimensionality reduction (20). The FindNeighbors function was then applied using these 20 dimensions, followed by cell clustering using the FindClusters function. Then, we used the FindAllMarkers to identify DEGs among different cell populations (21).

### 2.3 Analysis of fibroblast subpopulations

Fibroblasts were extracted from total cells, and then subjected to quality control, dimensionality reduction, clustering, and re-annotation using the Seurat (version 5.1.0).

### 2.4 Gene set activity score

To investigate the functional heterogeneity of fibroblast subpopulations, we used the AUCell algorithm for gene set enrichment analysis (22). Calculate the activity score of each fibroblast on the TGF-β gene set of MSigDB using the AUCell R package (version 1.30.1) (23).

### 2.5 Acquisition of epithelial-mesenchymal transition gene set

A comprehensive list of genes related to epithelial-mesenchymal transition was obtained from the dbEMT2.0 database (24), which provides a manually organized set of EMT related genes. We extracted all genes from it as the initial EMT gene pool.

### 2.6 Cellchat

We used the CellChat R package (v1.6.1) (25) to infer and visualize Intercellular communication networks based on annotated single-cell data. A CellChat object was generated from the Seurat object using cell cluster identities. The built-in human ligand–receptor database in CellChat was applied to identify overexpressed ligand–receptor interactions across cell populations.

To uncover the immunomodulatory pathways emanating from CAF subpopulations and their potential target cells, we utilized the NicheNet method (26).This method integrates prior knowledge of ligand-to-target signaling networks and predicts CAF-derived ligands that regulate gene expression programs in various recipient cell types. We conducted intercellular communication analysis among the eight major cell types identified in **Supplementary Figures 1A-S1D**.

### 2.7 Prognostic risk model

The feature genes of CAF_2 subgroup were intersected with the EMT gene set obtained from the dbEMT2.0 database to obtain 175 candidate genes for constructing a prognostic model. To prevent overfitting and select the most prognostic genes, we conducted LASSO-Cox regression analysis (27) using the glmnet R package (version 4.1-10) (28) on the TCGA-LUAD cohort. LASSO-Cox regression was run on the 175 candidate genes, with the optimal penalty parameter (λ) determined by the one-standard-error rule to ensure model parsimony (29). A refined signature of 16 genes was identified.

### 2.8 Risk score calculation

We use this equation to calculate the risk score of all samples in TCGA database:


Risk score=SHC1*0.172+ADM*0.053+CTSL*0.107+JAG1*0.107+NR2F2*0.159+CEBPB*0.069+LOX*0.016+BSG*0.078−HGF*0.202+TIMP1*0.083−NDRG2*0.034+CAV1*0.014+FERMT2*0.065−SLIT3*0.064−EGFR*0.010−PEBP1*0.110

### 2.9 Model validation

Stratification and survival analysis: Patients were dichotomized into high-risk and low-risk groups based on the median risk score. Kaplan-Meier survival curves were generated (30), and differences in overall survival between the two groups were compared using the two-sided log-rank test (31).The survival R package (version 3.8-3) (32)was used for this procedure. Time-dependent ROC analysis: The predictive accuracy of the risk score was assessed by time-dependent receiver operating characteristic (ROC) curve analysis using the timeROC R package (version 0.4) (33).

### 2.10 Construction of the prognostic nomogram

We employed rms R package (version 8.0-0) (34) to construct a nomogram following established methodological guidelines (35).This nomogram integrated the risk score with clinical parameters, including age, gender, and TNM stage. Calibration curves were utilized to evaluate the predictive performance of the nomogram.

### 2.11 Clinical relevance and external validation

Association of Risk Score with Survival Status: In the TCGA-LUAD dataset, the risk scores’ value between surviving and deceased patients at the last follow-up was compared, and statistical significance was evaluated using two-sided Wilcoxon rank sum test. Univariate Cox regression: we conducted a univariate Cox regression analysis to estimate the prognostic value of risk score and clinical parameters (36). External validation: Apply the risk score calculation formula and median cutoff value obtained from the TCGA cohort to the independent external dataset GSE72094 to test the robustness and generalizability of the risk score. Repeat survival analysis and nomogram calibration on this validation set.

### 2.12 Functional enrichment analysis

The limma R package (version 3.64.3) was utilized to find DEGs between high-risk and low-risk groups. Functional enrichment analysis of the DEGs was performed for Gene Ontology (GO) terms and Kyoto Encyclopedia of Genes and Genomes (KEGG) pathways using the clusterProfiler R package (v4.16.0) (37). Gene set enrichment analysis: To gain insights into broader biological pathways without relying on predefined DEG thresholds, we performed GSEA using the clusterProfiler package (38). We utilized the Hallmark gene set in MSigDB. The gene list was sorted based on the log~2~difference multiples obtained from DEG analysis.

### 2.13 Tumor microenvironment analysis

ESTIMATE algorithm: The ESTIMATE R package (39) (version 1.0.13) was employed to calculate ImmuneScore, StromalScore, and ESTIMATE Score for each sample. CIBERSORTx analysis: To quantify the proportion of 22 immune cell types, the CIBERSORTx algorithm (40) was applied to the TCGA-LUAD cohort, with analyses conducted based on the LM22 feature matrix and 100 permutations.

### 2.14 Prediction of immune therapy response

TIDE score assessment: The Tumor Immune Dysfunction and Exclusion (TIDE) algorithm (41) was utilized to calculate the score of every sample, so as to predict potential clinical responses to immune checkpoint blockade therapy.

### 2.15 RNA isolation and RT-qPCR

First, total RNA was extracted from the cells using TRIzol, and cDNA synthesis was performed using RT-qPCR Ver.2 (Accurate Biology, Hunan, China). The primer sequence is presented in **Supplementary Table 1**. Approximately 40 cycles were involved using the LightCycler^®^480 (Roche, USA). All these steps must strictly follow the manufacturer’s instructions.

### 2.16 Western blot analysis

Protein samples were extracted from cells treated with different methods and transferred onto NC membranes via SDS gel electrophoresis. The membranes were then blocked at room temperature using non-fat milk and incubated with the primary antibody for 16–18 hours. Subsequently, they were then washed three times with Tween 20 in Tris-buffered saline (TBST), followed by incubation with secondary antibodies at room temperature for 1–2 hours. Upon three repeated washes, protein expression levels were observed by adding chemiluminescence agents. The lung adenocarcinoma tissue samples were obtained from Jinan Central Hospital in Shandong Province, and their clinical medical records are shown in **Supplementary Table 2**. All patients provided written informed consent.

The antibodies used included anti-GAPDH (1:10000; Proteintech, 60004-1-Ig); anti-Vinculin (1:10000; Abways, CY5164); anti-NR2F2 (1:2000; ABclonal, A24647); anti-FAP (1:1000; ABclonal, A6349); and anti-α-SMA (1:50000; Proteintech, 80008-1-RR).

### 2.17 Human lung adenocarcinoma tissue microarray and immunohistochemistry

The process was initiated with dewaxing the paraffin sections and restoring antigenicity, followed by the addition of a 3% hydrogen peroxide solution. Finally, the sections were blocked with 3% BSA/serum. Add the primary antibody to the slices, incubate at 4 °C for 16–18 hours, and then stain with DAB, followed by counterstaining of the cell nucleus with hematoxylin. Then, dehydrate the film, scan the image, and analyze it using ImageJ. Herein, human lung adenocarcinoma tissue microarray (TMA) included human lung adenocarcinoma tissue specimens (n=15), and the corresponding normal specimens (n = 15) were purchased from Shanghai Xinchao Biological Research Co., Ltd. (Shanghai, China).

### 2.18 Immunofluorescence staining

Paraffin-embedded tissue sections were placed on a slide warmer at 60 °C for 2 hours. The sections were then dewaxed by immersion in xylene I and xylene II for 15 minutes each, followed by rehydration through a graded ethanol series (100%, 95%, 75%) for 5 minutes each, and finally rinsed with distilled water for 5 minutes. Antigen retrieval was performed using a pressure cooker: sections were immersed in EDTA antigen retrieval buffer (pH 9.0) and heated to boiling in a pressure cooker for 2 minutes, then allowed to cool naturally to room temperature. The sections were washed three times with phosphate-buffered saline (PBS), 5 minutes each wash. Subsequently, the sections were blocked with 5% bovine serum albumin (BSA) for 1 hour at room temperature. After removing the blocking solution, the sections were incubated with a mixture of primary antibodies overnight at 4 °C in a humidified chamber. Following three washes with PBS, the sections were incubated with fluorescence-conjugated secondary antibodies for 1 hour at room temperature in the dark. After three additional washes with PBS, the sections were mounted with mounting medium containing DAPI. Images were captured using a confocal laser scanning microscope under consistent acquisition parameters and processed accordingly.

### 2.19 Cell culture and transfection

The human cell lines A549 (English name: A549, stock number: ZQ0003) and H1299 (English name: NCI-H1299, stock number: ZQ0007) were purchased from the cell bank of Shanghai Zhongqiao Xinzhou Biotechnology Co., Ltd. (China Shanghai), while NAF (HFL1) and BEAS-2B were provided by the Cell Collection Network of the Chinese Academy of Sciences (China Shanghai). Disposable cell culture products such as cell culture dishes (Part No.: TCD010100) were purchased from Guangzhou Jet Bio-Filtration Co., Ltd. All cells were cultured in strict accordance with standard protocols. Cell culture medium media contained 10% fetal bovine serum and 1% penicillin/streptomycin. The cells were cultured and induced in a temperature-controlled chamber at 37 °C with 5% carbon dioxide.

The NR2F2 overexpression plasmid was purchased from Vigene Biosciences (China), and the empty pEnter vector was used as a negative control. CAFs or NAFs in the logarithmic growth phase were seeded into 6-well plates at a density of 2 × 10^5^ cells per well. Transfection was performed when the cells reached 50–60% confluence. The transfection reagent was used according to the manufacturer’s instructions. The plasmid DNA and transfection reagent were diluted separately in Opti-MEM medium (Gibco), incubated at room temperature for 5 minutes, mixed, and further incubated for 15 minutes to form transfection complexes. The complexes were added to the cells, and the medium was replaced with fresh complete medium 6 hours after transfection. Cells were collected 48 hours post-transfection, and the overexpression efficiency was validated by Western blot analysis of NR2F2 protein levels.

Three independent small interfering RNAs (siRNA) targeting the human NR2F2 gene and a negative control siRNA were designed and synthesized. All sequences are listed in **Supplementary Table 3**. Transfection was performed when the cell fusion rate reached 60-70%. After 48–72 hours, knockdown efficiency was verified through real-time quantitative PCR and Western blot.

### 2.20 Induction of cancer-associated fibroblast (CAF) phenotype

HLF1 cells were cultured until achieving 70-80% fusion rate, then replaced with fresh medium containing 10 ng/mL recombinant human TGF-β1. The control group consisted of untreated NAF cells cultured in parallel under identical conditions. The established CAF markers (including α-SMA and FAP) were validated by Western blot analysis to confirm the CAF-like phenotype. Hereafter, CAF-like cells induced by NAF cells will be collectively referred to as CAFs.

### 2.21 Co-culture system and functional assays

To investigate the mechanism by which NR2F2 expression levels in cancer-associated fibroblasts (CAFs) regulate lung adenocarcinoma cells, this study employed a Transwell co-culture system. CAFs with altered NR2F2 expression were subsequently seeded in the upper chamber (pore size 0.4 μm; Corning), while the lower chamber was inoculated with A549 or H1299 cells. The cells were co-cultured in complete medium. Subsequently, tumor cells in the bottom well were harvested, rinsed with PBS, and processed for downstream functional testing on cell proliferation and cell migration detection.

### 2.22 CCK-8 proliferation experiment

CCK-8 assays (Yeasen Biotechnology) were used to assess the proliferation of cancer cells. A549 and H1299 cells treated under various conditions were seeded in 96-well plates and cultured for 24, 48, 72, and 96 hours, respectively. Each well was supplemented with 10 µl of CCK-8 solution, followed by incubation at 37 °C for 1 hour. Absorbance measurements were taken using a spectrophotometer at 450 nm.

### 2.23 Wound healing assessment

Trypsin-digested cells were seeded into 6-well plates and cultured to full confluence. The cell monolayer was scratched with a pipette tip to create a cell-free gap, and the detached cells were then washed away. Cells were then rinsed three times with PBS, and the medium was immediately replaced with serum-free medium. Cultures were maintained in an incubator at 37 °C with 5% CO_2_, and images were captured using an inverted microscope (OLYMPUS, Japan) at both 0 and 24 hours.

### 2.24 EdU assays

Upon harvesting A549 or H1299 cells and seeding them in a 96 well plate, we added EdU (10μ M) to the cells and incubated (2 hours, 37 °C). After labeling, the cells were fixed with 4% paraformaldehyde, glycine, and other reagents. The fixed cells were treated with 0.5% Triton X-100, stained with the fluorescent dye for 10 minutes, and examined under a fluorescence microscope (Olympus, Japan).

### 2.25 Transwell migration and invasion assays

Cells were placed in the upper chamber of Transwell chambers and cultured in serum-free medium, while the lower chamber contained medium supplemented with 10% FBS. Following 24-hour incubation, the chamber cells were fixed with 4% paraformaldehyde and subsequently stained with crystal violet, with the cell counts recorded in five randomly selected areas.

For the invasion assay, Transwell inserts were pre-coated with Matrigel. Cells were seeded into the upper chamber in serum-free medium, while the lower chamber contained medium with 10% FBS as a chemoattractant. After 24 h, non-invaded cells on the upper surface were removed with a cotton swab. Cells that had invaded through the Matrigel to the lower surface were fixed with 4% paraformaldehyde, stained with crystal violet, and counted in five random fields per well.

### 2.26 Colony formation assay

Cells in the logarithmic growth phase were seeded into 6-well plates at a density of 500 cells per well, with three replicate wells per group. After cell attachment, the plates were cultured at 37 °C with 5% CO_2_ for 14 days, and the culture medium was replaced every 3 days. When visible colonies appeared, the medium was discarded, and the cells were gently washed twice with phosphate-buffered saline (PBS). Cells were then fixed with 4% paraformaldehyde for 30 minutes and stained with 0.1% crystal violet for 20 minutes. After washing with distilled water to remove excess stain, the plates were air-dried and photographed. Colonies containing at least 50 cells were counted using ImageJ software.

### 2.27 Statistical analysis

All quantitative data are presented as mean ± standard deviation and based on at least three independent experiments. For group comparisons, a two-tailed Student’s t-test was used, and a one-way ANOVA was initially performed for multiple comparisons, followed by a Tukey *post-hoc* test. A p-value below 0.05 is considered statistically significant. All analyses were conducted using the GraphPad Prism software package (version 8.0).

## 3 Results

### 3.1 Single cell identification of cell types

**Figure 1:**
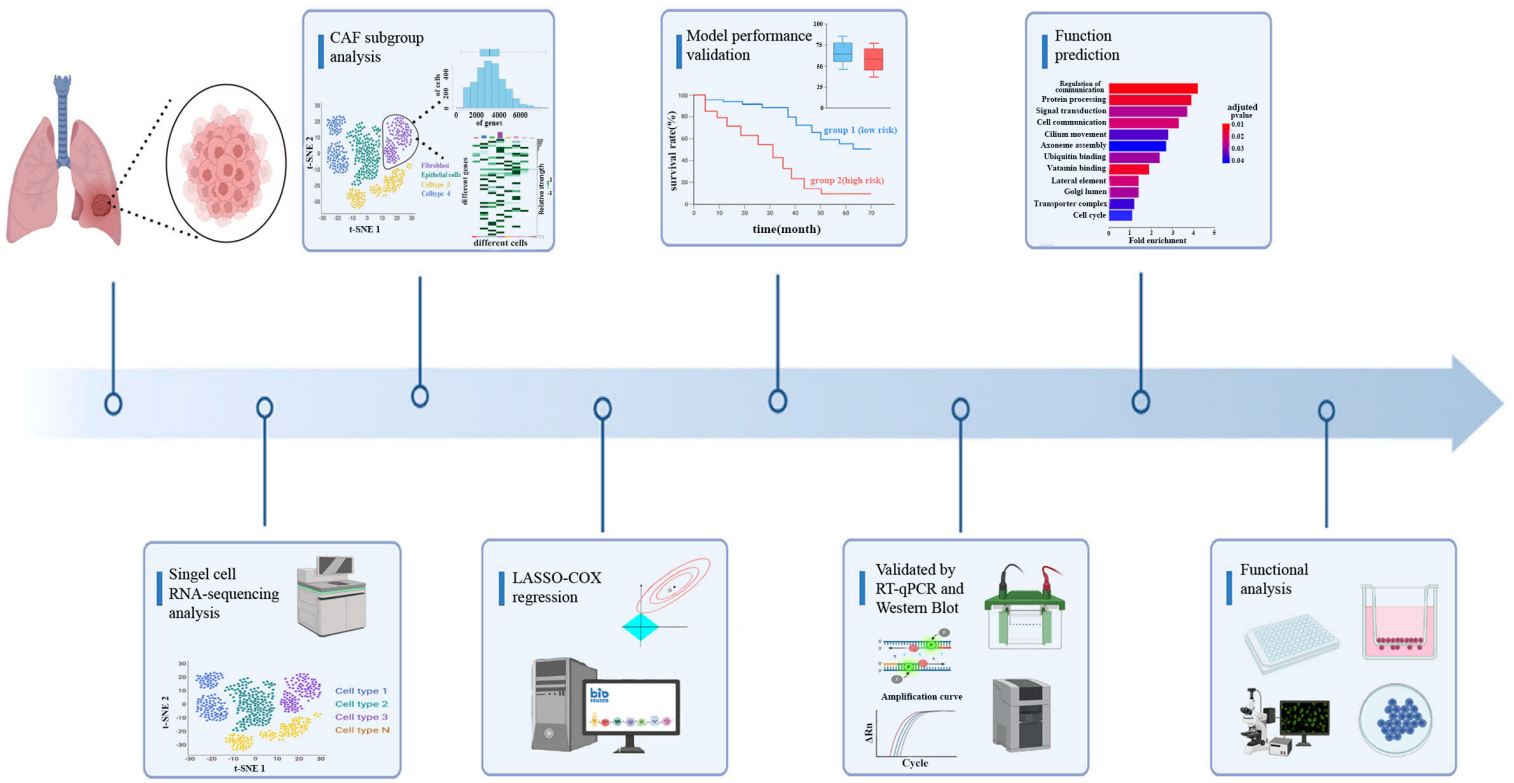
Schematic diagram of the experiment. Schematic representation of the study workflow, including the systematic identification and subsequent validation of hub genes.

**Figure 2:**
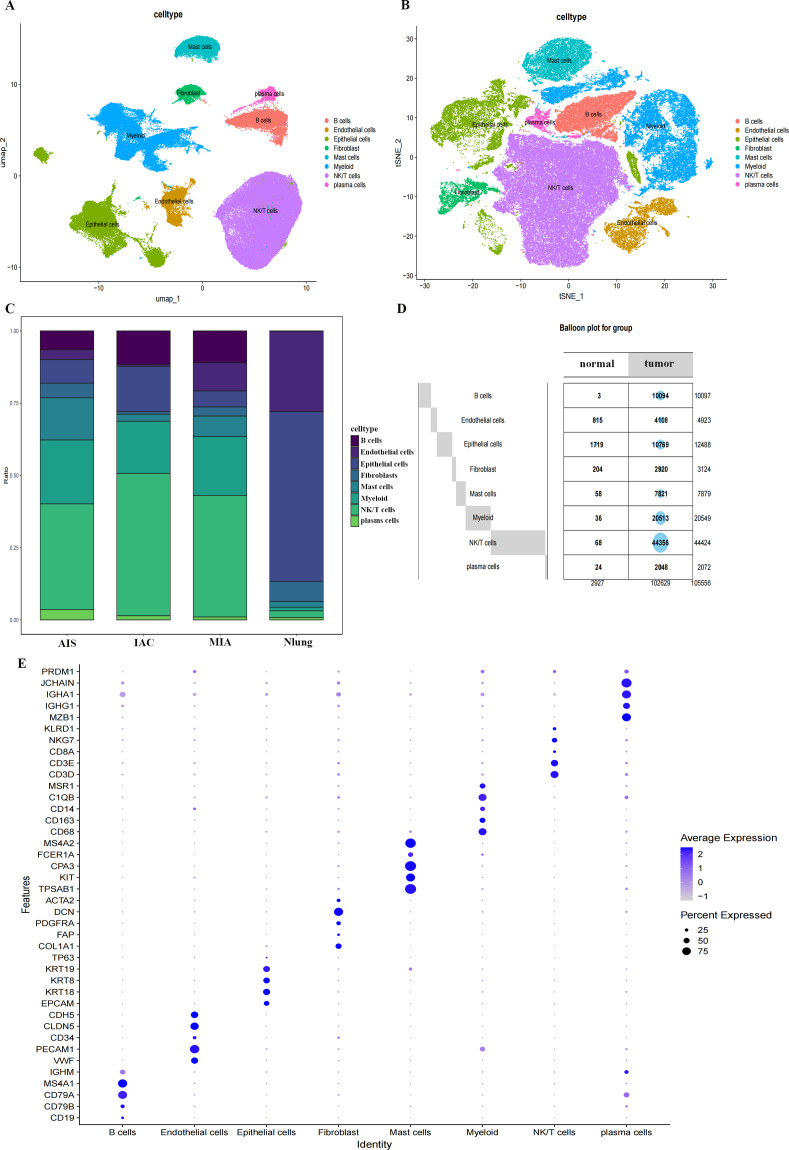
Single cell identification of cell types. **(A)** UMAP plot colored by cell type identity, as defined by expression of canonical cell type-specific markers. **(B)** t-SNE visualization of the same single-cell transcriptome data. **(C)** Stacked bar plot showing the proportional distribution of each cell type across the four sample groups (AIS, IAC, MIA, Nlung). **(D)** The balloon plot summarizes the absolute cell counts of each cell type in normal and tumor samples. **(E)** Dot plot showing the expression of key identified marker gene for cell type identification.

**Figure 1** presents the general flowchart. The quality control process of single-cell sequencing data is shown in **Supplementary Figure 2**. Successfully, batch effects were effectively mitigated using Harmony algorithm. PCA was first performed on the top 2000 hypervariable genes, and the dimensions were then reduced with t-SNE and UMAP (**Figures 2A, B**). The distribution of cell clusters across distinct groups is visualized in **Figure 2C**. Based on the cellular makeup among the samples, with the balloon plot showing the percentage of each kind of cell in every specimen, eight different types of cells were observed: NK/T cells(n =44424), B cells (n =10097), plasma cells (n =2072), mast cells (n =7879) Myeloid (n =20549), fibroblasts (n =3124), endothelial cells (n =4923), and epithelial cells (n =12488) (**Figure 2D**). A dot plot of canonical gene expression pattern corroborated cell type annotation (**Figure 2E**).

### 3.2 Identification of TGF-β-activated CAF subgroups with carcinogenic potential

To delve into the cellular composition of the immune microenvironment in lung adenocarcinoma, cell-cell communication analysis was hereby conducted for the 8 main cell types in **Supplementary Figures 1A−S1D**. Results revealed that fibroblasts were communicating strongly on the interaction network, demonstrating fibroblasts as rather active components of the tumor microenvironment.

**Figure 3:**
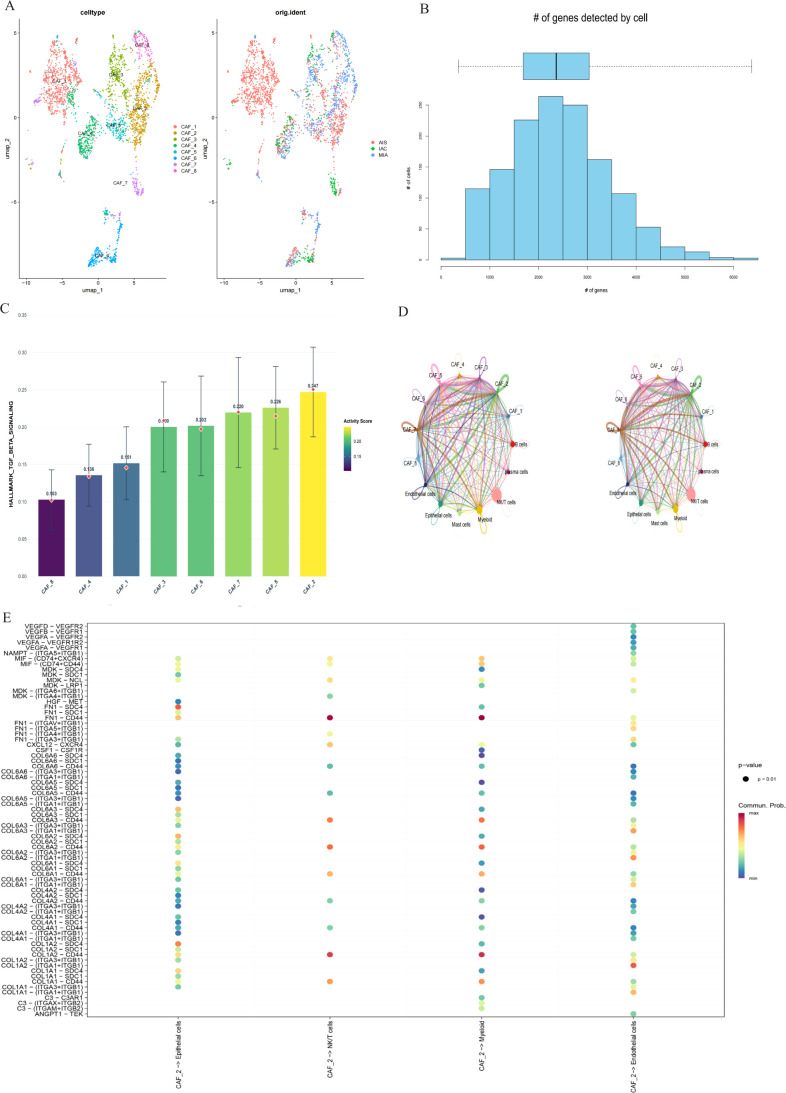
Identification of TGF-β activated CAF subgroups with carcinogenic potential. **(A)** UMAP visualization showing the identification of 8 distinct CAF subpopulations. **(B)** Bar chart shows a skewed distribution of gene expression in different cells. **(C)** Bar chart shows the activity of TGF-β signaling. **(D)** Chord diagram illustrating cell-cell communication between CAF subpopulations and other cell types. (left: number of interactions; right: interaction strength) **(E)** Bubble plot generated by netVisual_bubble showing communication probabilities between CAF_2 and other cell types.

On this basis, further endeavors were made to delve into the heterogeneity of the cancer-associated fibroblast population. Then, eight different types of CAF sub-clusters were identified (**Figure 3A**). The functional activity degree of each subcluster was subsequently evaluated using the AUCell algorithm (**Figure 3B**). Notably, CAF-2 displayed significant TGF-β signaling pathway activation (**Figure 3C**), confirming its possible involvement in reshaping the immune landscape.

To define intercellular communication amongst CAF subpopulations, specific cell-cell communication analysis was conducted (**Figure 3D**). Similarly, CAF-2 also exhibited a wide range of interaction profiles and engaged with considerable cell types via several ligand-receptor pairs such as CXCL12-CXCR4, HGF-MET, FN1-SDC1, and FN1-SDC4 (**Figure 3E**). These results verified the role of CAF-2 as an active signaling hub in the TME.

### 3.3 Construction and validation of a prognostic risk model based on CAF-2 signature genes

**Figure 4:**
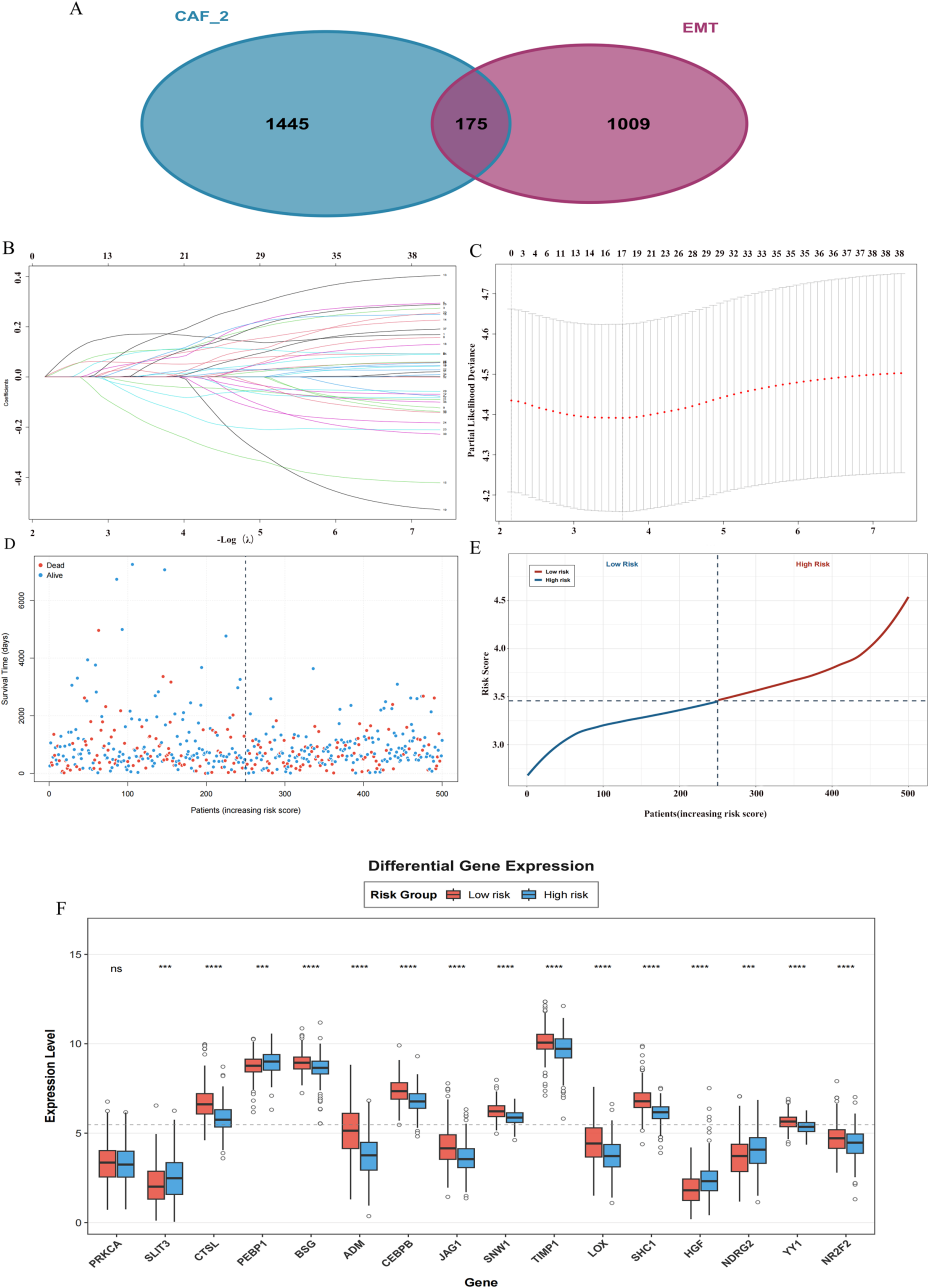
Construction and validation of a prognostic risk model based on CAF-2 signature genes. **(A)** The Venn diagram of CAF_2 subgroup characteristic genes and EMT, epithelial mesenchymal transition related genes. **(B)** LASSO-Cox regression analysis shows the coefficient contraction pathway of characteristic genes. **(C)** The curve of partial likelihood deviation as a function of LASSO model parameters (log (λ)). **(D)** Distribution of LUAD patients based on risk scores. **(E)** Distributions of OS status, OS and risk scores. **(F)** Gene expression in high and low prognostic models. ns, not significant; ***,p < 0.001, ****, p < 0.0001.

The activation of the TGF-β signaling pathway in CAF represents the core of poor patient prognosis, mainly through the formation of an immunosuppressive microenvironment and driving epithelial-mesenchymal transition (EMT) in tumor cells. Based on this hypothesis, the characteristic genes of CAF-2 and the EMT gene set were integrated for intersection analysis to accurately screen the core oncogenes involved in this process. In order to transform the significant gene overlap between CAF-2 and EMT (175 genes, **Figure 4A**) into a robust prognostic tool, LASSO-Cox regression was performed. Firstly, 175 genes were compressed into 41 prognostic related ones, which were then optimized to obtain the simplest feature set containing 16 genes. The optimal penalty coefficient λ was determined through the coefficient contraction path (**Figure 4B**) and the partial likelihood deviation curve (**Figure 4C**), ensuring both the simplicity and prediction accuracy of the model. Based on this last set of genes and their regression coefficients, the comprehensive risk score for each patient was calculated. Patients were stratified into high-risk and low-risk groups using the median risk score of the training cohort as the cutoff value (**Figure 4D**). Risk score served as an effective indicator for evaluating the level of sample risk (**Figure 4E**). Subsequently, the model genes were shown in the high-risk and low-risk subgroups (**Figure 4F**).

### 3.4 Clinical relevance and validation of prognostic models

**Figure 5:**
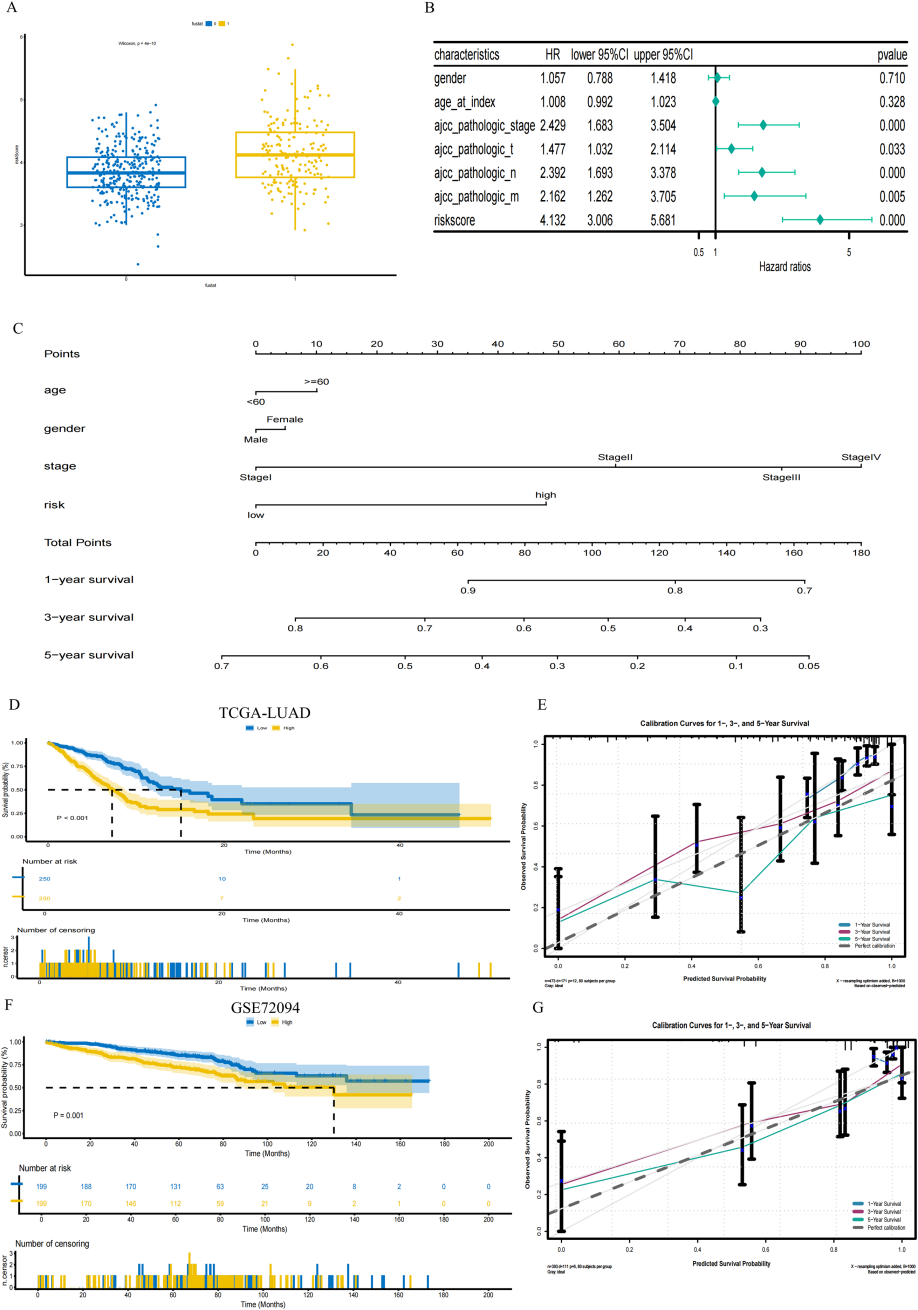
Validation of prognostic models and exploration of clinical relevance. **(A)** Box plot displaying risk scores for different groups (Wilcoxon test, p = 4e-10). **(B)** Forest plot of univariate Cox regression analysis for clinical characteristics and the risk score. **(C)** Nomogram is composed of age, gender, stage, and risk score to predict prognosis at 1, 3, and 5 years. **(D)** Kaplan-Meier survival curves for the training cohort (TCGA-LUAD). (Log-rank test, P < 0.001). **(E)** Calibration curves for the nomogram in the training cohort (TCGA-LUAD). **(F)** Kaplan-Meier survival curves for the external validation cohort (GSE72094). (Log-rank test, P = 0.001). **(G)** Calibration curves for the nomogram in the external validation cohort (GSE72094).

In the death group, the risk score was notably higher than that in the survival group (**Figure 5A**). Univariate Cox analysis also suggested that risk score was the strongest factor predicting the overall survival amongst clinicopathological factors (HR = 4.132, 95%CI: 3.006-5.681, p < 0.001) (**Figure. 5B**).

A nomogram including age, gender, and tumor stages was hereby developed (**Figure 5C**). As shown in **Figures 5D**, the survival of the low-risk patients in the TCGA-LUAD training set showed a significant difference compared with that of the high-risk patients. Calibration curves were quite consistent between predicted values and observations (**Figure 5E**).

External validation using the GSE72094 dataset confirmed the model’s robustness, involving distinct separation of survival curves (**Figure 5F**) and well-fitted calibration plots across time points (**Figure 5G**).

### 3.5 Differential transcriptomic profiling and enrichment analysis between risk groups.

**Figure 6:**
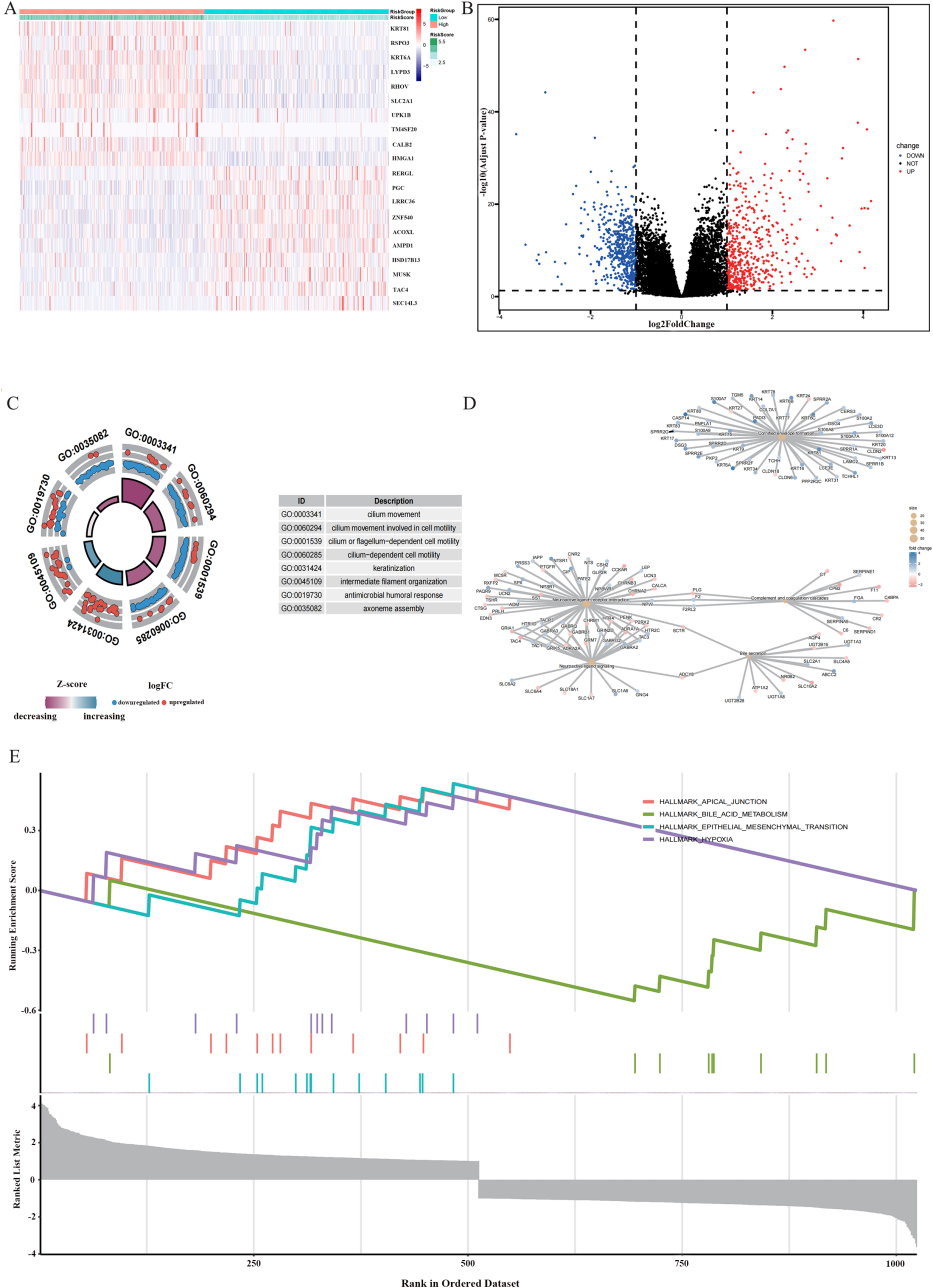
Differential transcriptomic profiling and enrichment analysis between risk groups. **(A)** Heatmap of significant differentially expressed genes. **(B)** Volcano plot of differentially expressed genes (DEGs). **(C)** Chord plot of Gene Ontology (GO) enrichment analysis. **(D)** Network diagram of KEGG pathway enrichment. **(E)** GSEA, Gene Set Enrichment Analysis plot.

To seek a more in-depth understanding of variations between different risky groups, transcriptomics analysis was conducted. Many DEGs were recognized between high- and low-risk patients (**Figures 6A, B**). Gene Ontology (GO) enrichment analysis revealed their involvement in significant biological processes, such as cell motility and ciliary function, structural remodeling and keratinization, immune-regulation, and protease regulation (**Figure 6C**). Following KEGG pathway analysis, the activation of keratinization, neuroactive ligand-receptor interaction, and metabolism-associated pathways in high-risk tumors was identified. These findings indicated abnormalities in differentiation, involvement of cells in active communications, and alterations in metabolisms for high-risk tumors (**Figure 6D**). Besides, the GSEA analysis showed that high-risk patients possessed high-enrichment for proliferation-related signaling like E2F targets, G2M checkpoint, EMT and metabolic signatures (**Figure 6E**). The results suggested that tumors in the high-risk group exhibited more coordinated biological programs. In this situation, tumors possessed more robust proliferative capacity and could metastasize more easily and better adapt to metabolism changes.

### 3.6 High-risk score associated with an immunosuppressive microenvironment and adverse immunotherapy response

**Figure 7:**
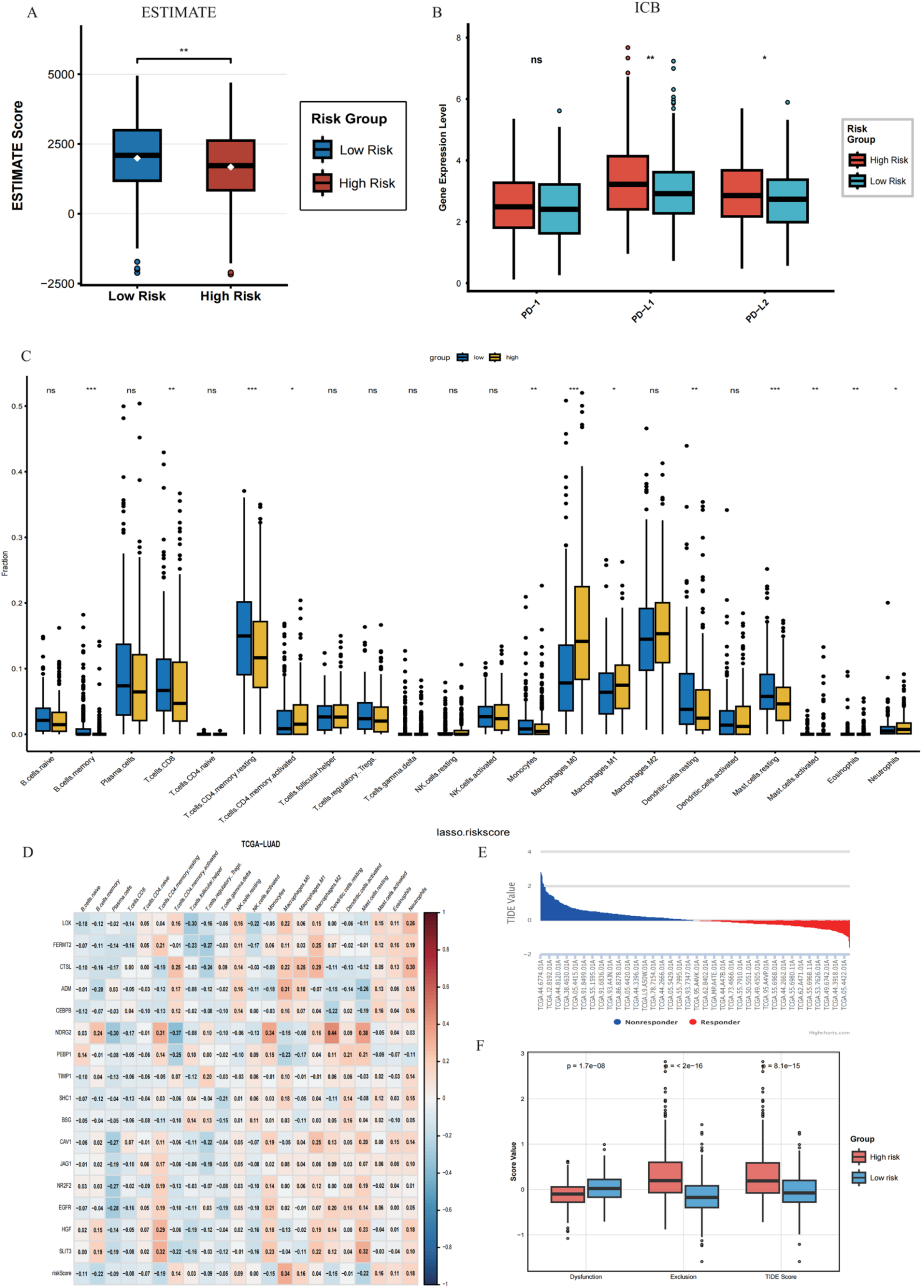
High-risk score associated with an immunosuppressive microenvironment and adverse immunotherapy response. **(A)** The box diagram shows the ESTIMATE score between the high-risk group and the low-risk group. **(B)** The expression levels of key immune checkpoint molecules (PD-1, PD-L1, PD-L2) between high-risk and low-risk groups. **(C)** The infiltration ratio of 22 immune cell subtypes in the high-risk and low-risk groups analyzed using the CIBERSORT algorithm (box plot). **(D)** The correlation heatmap between the core genes, risk score, and 22 immune cell infiltration levels of the prognostic model. **(E)** Bar chart displaying TIDE values for each sample. **(F)** The box diagram shows the TIDE score, T cell rejection score, and T lymphocyte dysfunction score between the high-risk and low-risk groups. *, p < 0.05; **, p < 0.01; ***, p < 0.001.

To clarify to the link between these prognostic models and the TIME features, microenvironment score analysis was carried out using ESTIMATE algorithm on the TCGA-LUAD queue. Results indicated that the ESTIMATE score among the high-risk patients was lower than that of the low-risk group, indicating much lower tumor immune infiltration level of the high-risk group compared with the low-risk group (**Figure 7A**).

The importance of immune checkpoint blockade therapy in lung cancer is widely acknowledged. Consequently, three main immune checkpoint molecules were further compared, and PD-L1 expression was observed to be significantly upregulated in the high-risk group (**Figure 7B**).

To further study the distribution of immune cells in the body, the CIBERSORT deconvolution software was employed to obtain the infiltration ratio of 22 types of immune cell subgroups. The high-risk group exhibited more immune-suppressive factors, characterized by a higher abundance of pro-tumor M0 macrophages. The proportion of cells with the potential to fight cancer, e.g., plasma cells and the resting form of CD4+ memory T cells, was reduced (**Figure 7C**).

Furthermore, analyses were also conducted over the correlation between the 16 genes included in the risk model, risk scores, and the infiltration levels of various immune cells through relevant heat maps (**Figure 7D**). Results showed that risk scores were positively correlated with M0 macrophages, yet negatively correlated with various anti-tumor immune cells such as plasma cells.

Finally, based on the TIDE database, the scores of T cell rejection, T cell depletion, and TIDE between the two groups of patients were evaluated (**Figures 7E, F**), confirming the poor response of the high-risk group to immune checkpoint inhibitor therapy.

In summary, this study systematically reveals that high-risk lung adenocarcinoma patients exhibit tumor microenvironment characteristics dominated by immune suppression, in which immune cells such as M0 macrophages may play a key role.

### 3.7 Prognostic validation of model genes

**Figure 8:**
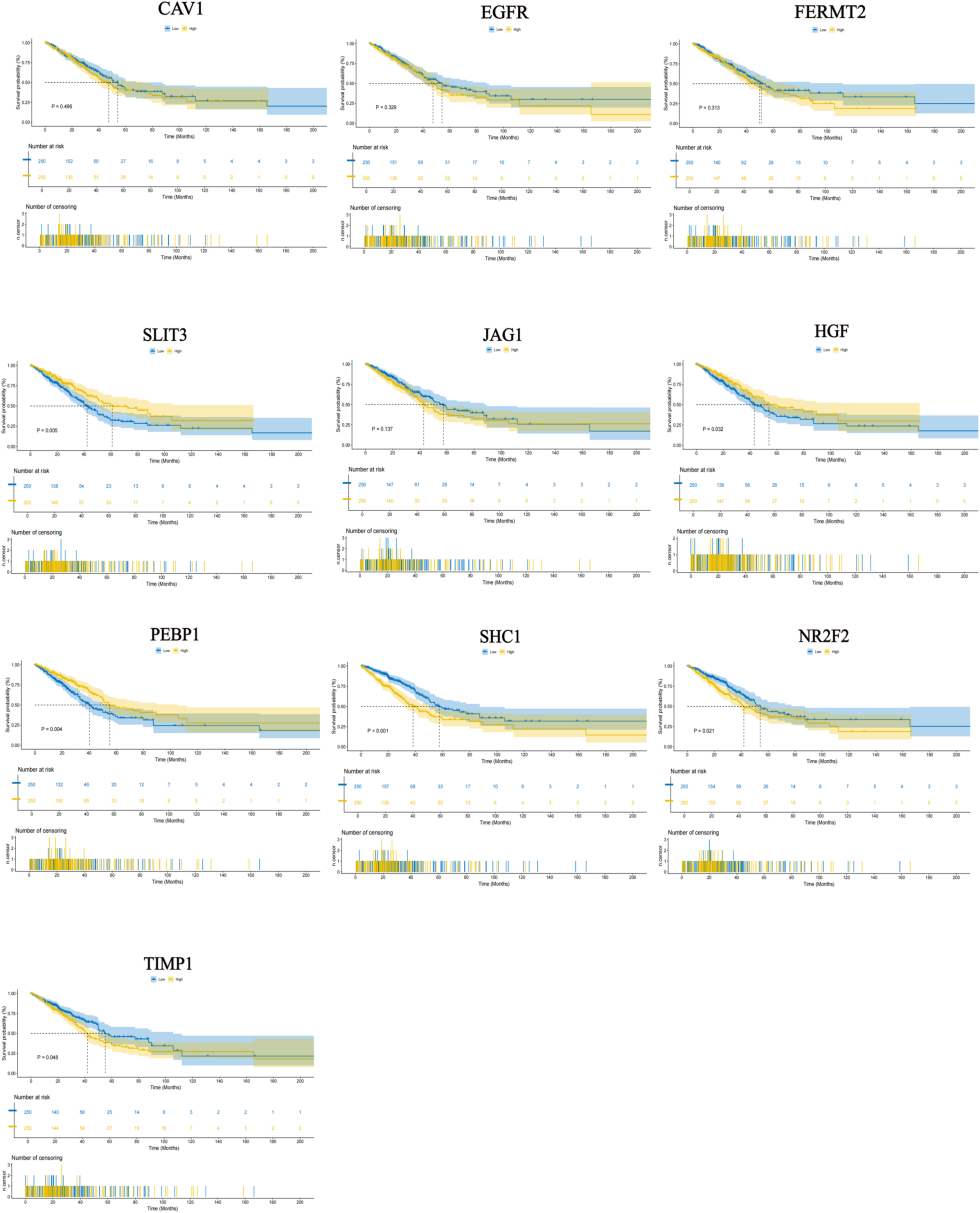
Prognostic validation of model genes. Prognostic validation of ten hub genes through Kaplan-Meier survival analysis.

Firstly, in order to validate the effectiveness of the model and identify core oncogenes, 10 genes with high coefficients were selected for validation (**Figure 8**). Consistent with the model’s predictions, high expression of SLIT3, HGF, and PEBP1 was associated with favorable patient survival, whereas high expression of TIMP1, SHC1, and NR2F2 was associated with poor survival. Among these genes, HGF, PEBP1, TIMP1, SHC1, and NR2F2 contributed the most to the proposed model, suggesting that these genes could indicate the prognosis and might become important prognostic biomarkers for LUAD patients.

### 3.8 Validation of model genes and preliminary exploration of core gene NR2F2

**Figure 9:**
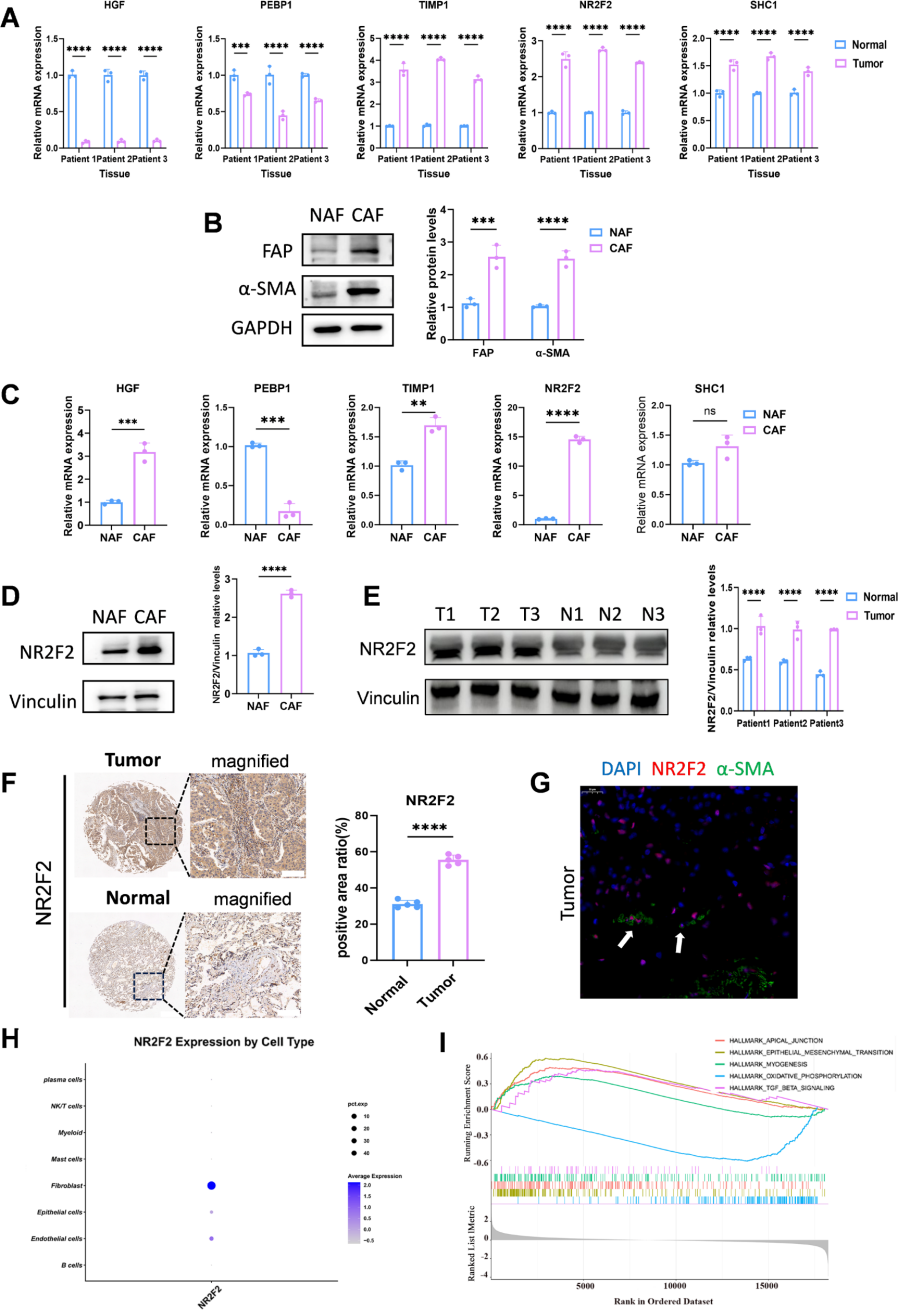
Validation of model genes and preliminary exploration of core gene NR2F2. **(A)** Transcriptional levels of HGF, PEBP1, TIMP1, NR2F2, and SHC1 in LUAD tissues relative to surrounding non-cancerous tissues were validated using RT-qPCR. **(B)** Successful induction was confirmed by Western blot analysis of CAF activation markers. **(C)** Differential expression of HGF, PEBP1, TIMP1, NR2F2, and SHC1 between CAFs and NAFs was detected by RT-qPCR. **(D)** Basal expression levels of NR2F2 in CAFs and NAFs were measured by Western blot. **(E)** Comparative Western blot results of NR2F2 expression in LUAD tissues versus surrounding healthy tissues. **(F)** Representative IHC staining images of NR2F2 in TMA, tissue microarray cohort (Scale bars: upper panel, 200 µm; lower panel, 50 µm). **(G)** Immunofluorescence staining images of α-SMA (green), NR2F2 (red), and DAPI (blue) in lung adenocarcinoma tissue sections (scale: 25 µm). **(H)** Dot plot analysis of NR2F2 expression levels across different cell types. **(I)** GSEA enrichment analysis revealed signal-enriched pathways of NR2F2. n ≥ 3, **p < 0.01, ***p < 0.001, **** p < 0.0001.

The transcriptional expression differences of these five genes in LUAD tissues were further explored by RT-qPCR. Evidently, the HGF and PEBP1 expressions were reduced in the LUAD tissues, while the NR2F2, SHC1, and TIMP1 expressions were elevated (**Figure 9A**). In addition, expressions of these five genes at the cellular level were investigated. We used single-cell sequencing technology to investigate the expression of NR2F2 in fibroblasts in tumor and normal samples (**Supplementary Figure 3**), and found that the expression of NR2F2 in cancer-associated fibroblasts was much higher than that in normal fibroblasts. Furthermore, NAFs was treated with TGF-β1 to induce cancer-associated fibroblasts (CAFs), and Western blot was used to detect relevant indicators of cancer-associated fibroblasts to verify the successful induction (**Figure 9B**). The CAFs used in subsequent experiments were induced using this method, and their successful induction was validated. Upon obtaining the CAFs, RT-qPCR was employed to detect the expression differences of 5 genes in NAFs cells and CAFs. In CAFs, the expression levels of NR2F2 and TIMP1 were significantly higher than those in NAFs cells; SHC1 showed no significant difference between CAFs and NAFs cells, while the most significant difference was observed in NR2F2 gene expression (**Figure 9C**). Based on these results and previous prognostic analyses, oncogenic roles of NR2F2 were further explored. Beyond the level of mRNA, changes in the protein expression were also detected using Western blot. The banding results indicated that compared with NAFs, the expression of NR2F2 was significantly increased in CAFs (**Figure 9D**). We used BEAS-2B cells for further validation, and the results were consistent with our expectations (**Supplementary Figure 4**). Then, Western blot was further employed to verify the expression levels of NR2F2 in the three pairs of LUAD tissues and their corresponding adjacent tissues not cancerous (**Figure 9E**). To determine NR2F2’s expression in human lung adenocarcinoma and elucidate its clinical implication, TMA was utilized for immunohistochemical analysis. According to the statistical data analysis, the expression level of NR2F2 in lung adenocarcinoma tissues was significantly enhanced compared with the corresponding non-cancerous tissues (**Figure 9F**). Subsequently, we observed the co-localization of NR2F2 with the CAF marker α-SMA using immunofluorescence staining. The results showed that in CAF, the fluorescence intensity of α-SMA (green) was significantly enhanced and was mainly located in the cytoplasm, while NR2F2 (red) was pointedly enriched in the nucleus. The Merge image demonstrated that within the same cell, both nuclear NR2F2 and cytoplasmic α-SMA signals were present simultaneously, indicating that they were co-expressed in CAFs (**Figure 9G**). These results confirmed the presence and upregulation of NR2F2 in lung adenocarcinoma. To further investigate the oncogenic mechanism of NR2F2, single-cell sequencing was analyzed to observe the expression levels of NR2F2 in different cells, and it was observed that NR2F2 presented the highest expression level in CAFs (**Figure 9H**). Enrichment analysis was also performed on NR2F2 through transcriptome sequencing, indicating that tumors with high NR2F2 expression had increased EMT activity, higher activity in the myogenesis and TGF-β pathway, and elevated apical membrane junction pathway activity. Besides, oxidative phosphorylation was inhibited (**Figure 9I**). This indicated that high expression of NR2F2 in CAFs promoted tumor invasion, matrix remodeling, and metabolic reprogramming.

### 3.9 Co-culture with NR2F2 overexpression CAFs enhances the proliferation, migration and invasion ability of tumor cells

**Figure 10:**
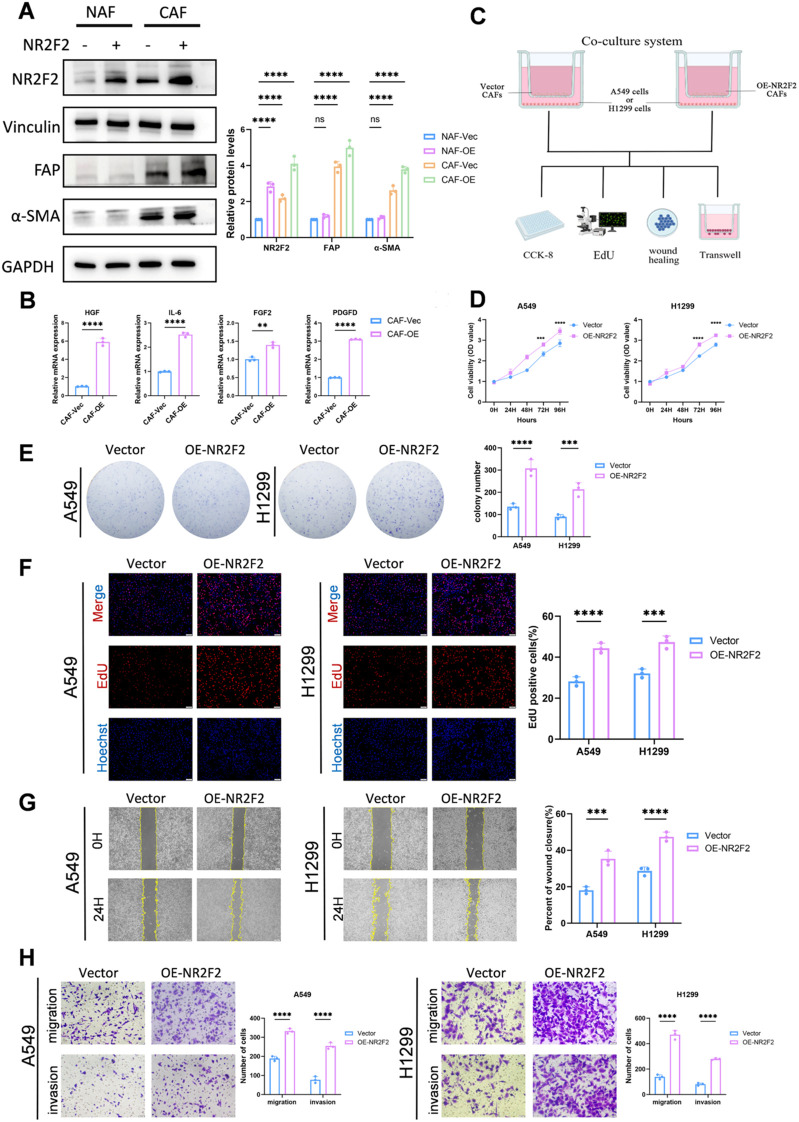
Co-culture with NR2F2 overexpression CAFs promote the proliferation, migration and invasion ability of tumor cells. **(A)** Expression levels of α-SMA, FAP, and NR2F2 proteins in NAFs, normal-associated fibroblasts and CAFs,cancer-associated fibroblasts were analyzed by Western blotting and quantitative detection under specific conditions. **(B)** Relative mRNA expression levels of HGF, IL-6, FGF2, and PDGFD were detected by RT-qPCR in CAF-Vec and CAF-OE groups. **(C)** Schematic diagram of co-culture system between CAFs and tumor cells. **(D)** Proliferation efficiency was validated by CCK8 assay. **(E)** Proliferation efficiency was evaluated by colony formation assay. **(F)** Proliferation efficiency was confirmed by EdU assay (scale: 100 µm). **(G)** Proliferation efficiency was assessed by wound healing experiment (scale: 400 µm). **(H)** Migration and invasion capabilities were verified by Transwell assay (scale: 200 µm). n ≥ 3, **p < 0.01, ***p < 0.001, **** p < 0.0001.

To investigate the role of NR2F2 in cancer-associated fibroblasts (CAFs), we modulated NR2F2 expression in normal associated fibroblasts (NAFs) and CAFs. Successfully overexpressing cells were designated as NAF-OE and CAF-OE, while cells transfected with the empty vector were designated as NAF-Vec and CAF-Vec. Western blot analysis revealed that NR2F2 overexpression alone did not upregulate the expression of CAF markers α-SMA and FAP in NAFs, indicating that NR2F2 overexpression alone is insufficient to convert NAFs into a CAF phenotype. In contrast, in CAFs that had been pre-induced by TGF-β, NR2F2 overexpression significantly enhanced their activation markers (**Figure 10A**). These data suggest that although NR2F2 alone cannot initiate the conversion of NAFs to CAFs, it plays a synergistic role in enhancing the activation of cancer-associated fibroblasts and maintaining their activated state in the presence of initiating signals such as TGF-β.

We next sought to explore how this enhanced activation state is achieved. Based on the literature, we hypothesized that NR2F2 enhances CAF function by regulating their paracrine signaling. To test this, we evaluated the expression levels of pro-inflammatory and pro-tumorigenic cytokines in CAFs using real-time quantitative PCR (RT-qPCR). Consistent with the enhanced activation state, NR2F2-overexpressing CAFs exhibited significantly elevated mRNA levels of HGF, IL-6, FGF2, and PDGFD compared to control CAFs (**Figure 10B**). These results suggest that NR2F2 not only enhances the contractile function (α-SMA) and matrix remodeling capacity (FAP) of CAFs but also amplifies their secretory phenotype, potentially contributing to the formation of a more pro-tumorigenic microenvironment.

Previous studies have shown that there is strong intercellular communication between cancer associated fibroblasts (CAFs) and lung adenocarcinoma (LUAD) cells, mainly mediated by pro neural signaling pathways such as epithelial mesenchymal transition (EMT) and transforming growth factor beta pathways. So, we further explore how NR2F2 regulates CAF paracrine function, thereby affecting the tumor microenvironment. Our follow-up research aims to explore the mechanism by which overexpression of NR2F2 in CAFs promotes the malignant behavior of LUAD cells, including proliferation, migration, and invasion.

To this end, a co-culture system based on Transwell technology was established to enable interaction between CAFs and LUAD cells (A549 and H1299). A549 and H1299 cells were co-cultured with CAFs transfected with either control plasmid (Vector) or NR2F2 overexpression plasmid (OE-NR2F2), followed by subsequent functional assays (**Figure 10C**). CCK-8 assays revealed that NR2F2 overexpression in CAFs significantly enhanced the proliferation of both A549 and H1299 cells upon co-culture (**Figure 10D**). Colony formation experiments further confirmed that the results of the clone formation experiments showed that the number of clones formed by A549 and H1299 cells co-cultured with NR2F2 overexpressing CAFs was significantly increased compared with the control group, indicating that the expression of NR2F2 in CAFs can enhance the long-term proliferation potential and clonogenicity of lung adenocarcinoma cells, further supporting its tumor-promoting effect (**Figure 10E**). EdU assays further corroborated that CAFs overexpressing NR2F2 can promote tumor growth (**Figure 10F**).

Given the importance of tumor cell migration and invasion in tumor progression and metastasis, wound healing and Transwell assays were performed on tumor cells co-cultured with NR2F2-overexpressing CAFs. Wound healing assays showed that compared with the control group, co-culture with CAFs overexpressing NR2F2 significantly increased the number of A549 and H1299 cells migrating into the wound area (**Figure 10G**). Moreover, Transwell assays demonstrated that NR2F2 overexpression markedly enhanced the migration and invasion capacities of tumor cells (**Figure 10H**). Consistent with these findings, repeated experiments with NR2F2 knockdown reduced the malignant potentials of LUAD cells, including proliferation, migration, and invasion (**Supplementary Figure 5**).

These results collectively indicate that overexpression of NR2F2 in CAFs significantly promotes the malignant phenotype of LUAD cells, whereas knockdown of NR2F2 effectively reverses its pro-oncogenic effects, providing robust evidence for NR2F2 as a potential therapeutic target in lung adenocarcinoma.

## 4 Discussion

Lung adenocarcinoma remains a fatal disease with high heterogeneity and complicated TME (42). Systematic analysis was hereby carried out on the cellular ecosystem of LUAD, with special emphasis paid on the differentiation of CAFs. This work shows following key findings: (1) Identification of a TGF-β-high CAF subpopulation (CAF-2) and construction and validation of a predictive model based on the intersection of CAF-2 marker genes with EMT genes. (2) Identification and function validation, suggesting that NR2F2 plays an important role in CAFs, which not only regulates CAF-mediated matrix remodeling but also promotes tumor progression.

The present scRNA-seq analysis shows that as previously reported, there is widespread heterogeneity within the LUAD CAF population (43, 44). CAFs are not a homogeneous population, and its heterogeneity is key to the complexity of TME and treatment resistance (45). In recent years, studies have used single-cell technology to classify CAFs into multiple functional subtypes, such as myofibroblast-like CAFs (myCAFs), inflammatory-like CAFs (iCAFs), and antigen-presenting CAFs (apCAFs) (46, 47). The CAF-2 subgroup is characterized by high expression of both matrix remodeling and immune regulation related genes, with high activation of the TGF-β signaling pathway. TGF-β signaling is the core driving factor modulating the phenotype and function of CAFs, mainly pushing them towards an “activated state” with strong profibrotic, pro-invasive, and immunosuppressive properties (4, 48). This is highly consistent with the present findings: high-risk patients in the CAF-2 feature model exhibit significant matrix remodeling, reduced CD8+T cell infiltration, and high PD-L1 expression in their TME. Therefore, CAF-2 may represent a specialized subgroup of CAFs with the capacity to drive an immunosuppressive TME, which partially explains the ineffectiveness of simple immune checkpoint inhibitors in some LUAD patients (49).

A 16 gene prognostic model was established and transformed into a clinically translatable one. The high scores of the TCGA training queue and the independent external validation queue (GSE72094) further demonstrated the robustness of the model. Notably, a high-risk score was clinically significant, as it not only correlated with poor patient survival but was also associated with more invasive tumor subtypes. It was associated with immunosuppressive TME, reduced TME infiltration, increased macrophages, and decreased infiltration of resting CD4+memory T cells, and plasma cells. PD-L1 was significantly upregulated in high-risk patients, indicating that these patients might benefit from combined immune checkpoint blockade therapy in an immunosuppressive environment, providing an option for patient classification (50).

NR2F2 represents a transcriptional regulatory molecule. Previous studies have mainly focused on the impact of its expression in tumor cells on tumor phenotype, and found that its promotion or inhibition function is related to changes in tumor type and microenvironment. For example, contrasting with previous reports that identified NR2F2 as a transcription factor that could directly promote cancer growth such as skin cancer and oral squamous cell carcinoma (51, 52). Our research indicates that NR2F2 can induce various phenotypic changes in tumors by regulating the expression patterns of CAFs. Our WB results show that overexpression of NR2F2 does not transform NAFs into CAFs, but rather plays a synergistic role in promoting the function of CAFs, which has been further confirmed. When co cultured with tumor cells, overexpression of NR2F2 in CAFs significantly promotes the proliferation, migration, and invasion potential of tumor cells. These results clearly indicate that NR2F2 becomes a powerful driving force for tumor invasiveness by enhancing key malignant phenotypes. Overall, this study provides groundbreaking evidence that NR2F2 can regulate CAF in TME, thereby shaping the immunosuppressive microenvironment and promoting tumor growth and invasion. These findings provide new insights into the complex immune microenvironment of lung adenocarcinoma and the regulatory mechanisms of CAF mediated niche remodeling.

Despite these findings, there are still some limitations worth considering in current research. The precise molecular mechanisms by which NR2F2 exerts its unique functions in the matrix and epithelium, such as specific target genes and co factors, still need to be fully elucidated. However, our research and several pieces of evidence from existing literature provide clues to the potential pathways involved. Firstly, our scRNA-seq and Bulk-seq analysis showed that NR2F2 high CAF was significantly enriched in the TGF - β signaling pathway and EMT related gene clusters. Given that TGF - β signaling is widely recognized as a major regulator of CAF activation and matrix remodeling (53), NR2F2 may work synergistically with TGF - β to maintain the activated CAF phenotype. Secondly, our gene set enrichment analysis identified a strong association between NR2F2 expression and extracellular matrix remodeling genes, indicating that NR2F2 may directly or indirectly regulate the expression of matrix components or matrix modifying enzymes such as collagen and matrix metalloproteinases. Thirdly, previous research reports have stated that NR2F2, as a transcription factor (54), can regulate the expression of genes involved in cell differentiation and tissue remodeling. Based on these observations, we speculate that NR2F2 in CAF may transcriptionally regulate key mediators of TGF - β/Smad axis or EMT related cytokines, thereby amplifying paracrine signals driving tumor cell invasion. Future research will use chromatin immunoprecipitation sequencing (Chip-seq) and transcriptomic analysis of NR2F2 manipulated CAF, which is crucial for identifying its direct transcriptional targets and comprehensively delineating downstream cascades.

In summary, this comprehensive multi-omics study describes the heterogeneity dimension of LUAD, identifies CAF-2 subgroup with high TGF-β activity, and translates this finding into a robust prognostic feature. More importantly, it also validates the carcinogenic effect of NR2F2 through transcriptome data and functional experiments: it exerts a remodeling function in the matrix, thereby regulating the proliferation and migration of tumor epithelial cells. This dual function positions NR2F2 as a central node connecting matrix evolution and tumor cell migration. It provides a novel theoretical framework for understanding TME interactions and laying a solid foundation for advancing NR2F2 as a promising therapeutic target to overcome tumor matrix-driven progression.

## Data Availability

The original contributions presented in the study are included in the article/**Supplementary Material**. Further inquiries can be directed to the corresponding authors.

## Supplementary material

Supplementary Figure 1 Interaction strength **(A)** and number **(B)** among different cells. Heatmap of outgoing signaling patterns showing cell sending signal strength (left)**(C)**and total signaling strength (right) **(D)**.

Supplementary Figure 2 Quality control of scRNA-seq data.

Supplementary Figure 3 Expression of NR2F2 in fibroblasts (scRNA-seq).

Supplementary Figure 4 **(A)** Expression of 5 candidate genes in BEAS-2B and B-CAFs **(B)** WB display of NR2F2 expression levels in BEAS-2B and B-CAFs. **(C)** Expression levels of CAF activation markers induced by BEAS-2B.n ≥ 3, *p < 0.05, **p < 0.01, ***p < 0.001, **** p < 0.0001.

Supplementary Figure 5 Co-culture with NR2F2 knockdown CAFs reduces the proliferation, migration and invasion ability of tumor cells. **(A)** Schematic diagram of co-culture system of CAFs and tumor cells. **(B)** Efficiency validation of NR2F2 knockdown using RT-qPCR. **(C)** Efficiency validation of NR2F2 knockdown using Western Blot. **(D)** CCK8 experiment verifies proliferation efficiency. **(E)** EdU experiment verifies proliferation efficiency. (scale bar:200 µm) **(F)** Wound healing assessment verifies proliferation efficiency. (scale bar:400 µm) **(G)** Transwell assay verifies migration and invasion ability. (scale bar:200 µm) n ≥ 3, *p < 0.05, **p < 0.01, ***p < 0.001, **** p < 0.0001.

Supplementary Table 1 Primer sequences for RT-qPCR experiments.

Supplementary Table 2 De-identified clinical data of lung adenocarcinoma patients.

Supplementary Table 3 Si-RNA sequences.

## Glossary

scRNA-seq: single-cell RNA sequence
EMT: epithelial-mesenchymal transition
CAFs: cancer-associated fibroblasts
NAFs: normal-associated fibroblasts
GO: Gene Ontology
KEGG: Kyoto Encyclopedia of Genes and Genomes
LUAD: lung adenocarcinoma
RTq-PCR: Reverse transcription quantitative real-time PCR
RNA-seq: RNA-sequencing
TCGA: The Cancer Genome Atlas
TME: tumor microenvironment
GEO: Gene Expression Omnibus.
