# Supplementary material for: NR2F2 in cancer-associated fibroblasts drives immune microenvironment remodeling and promotes lung adenocarcinoma progression
Source: Front Immunol. 2026 Apr 13;17:1776008. doi: 10.3389/fimmu.2026.1776008 (PMC13110960; doi:10.3389/fimmu.2026.1776008)

Full unedited blot for Figure 9B

marker 26616

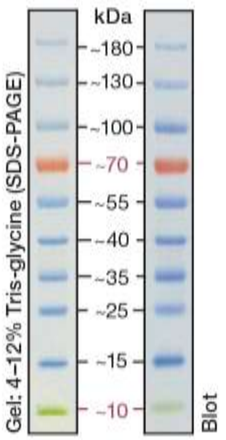

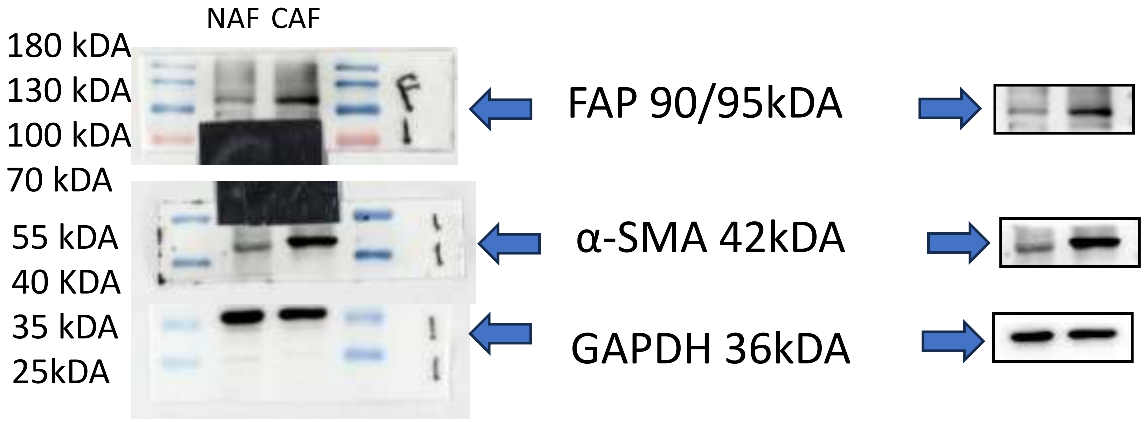

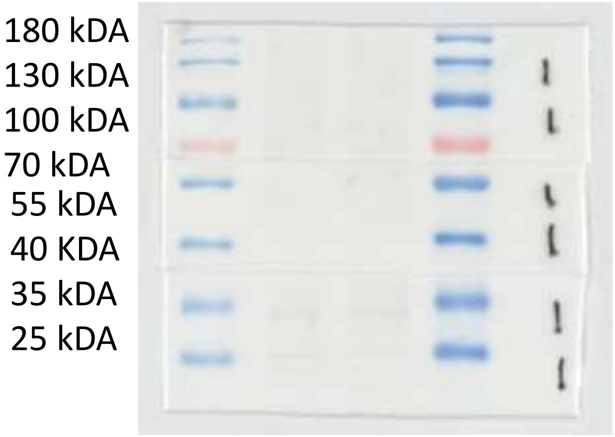

Full unedited blot for Figure 9D

marker 26616

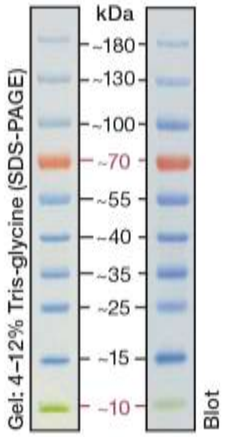

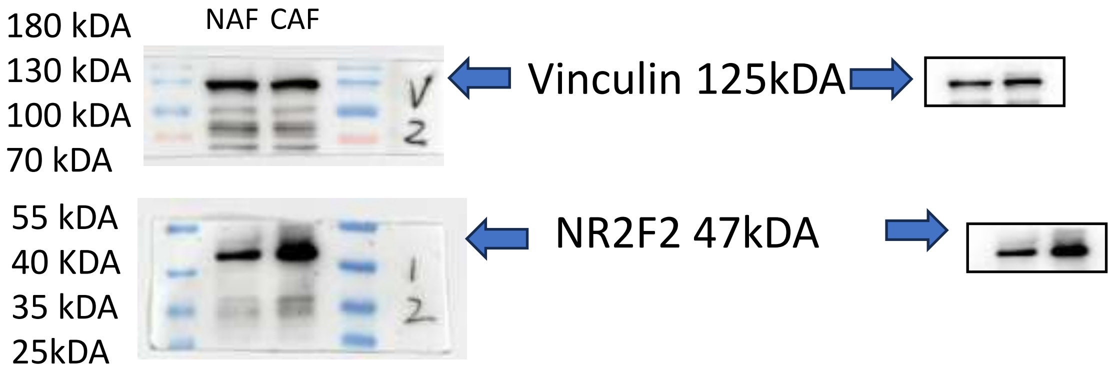

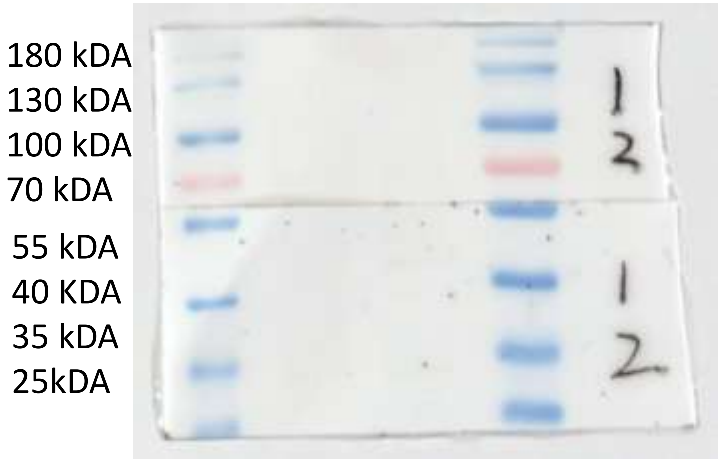

Full unedited blot for Figure 9E

marker 26616

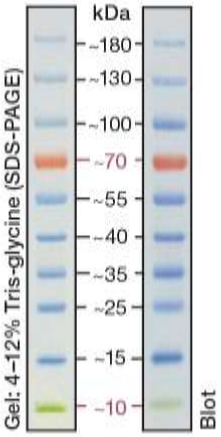

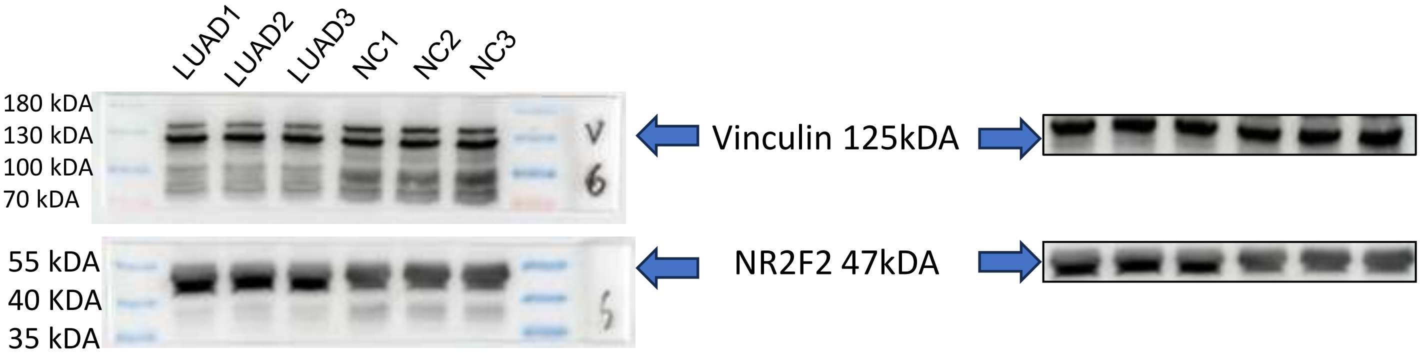

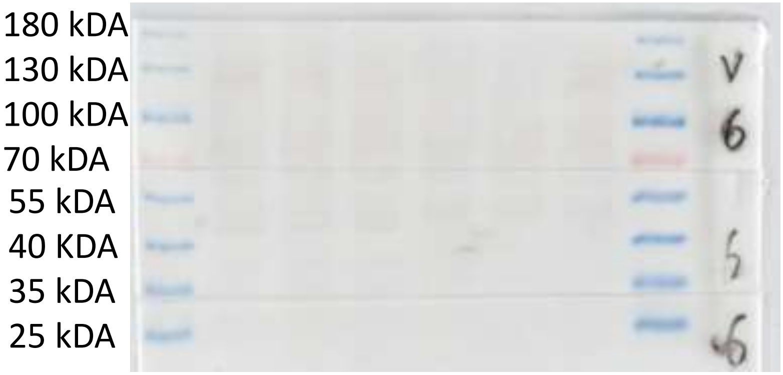

Full unedited blot for Figure 10A

marker 26616

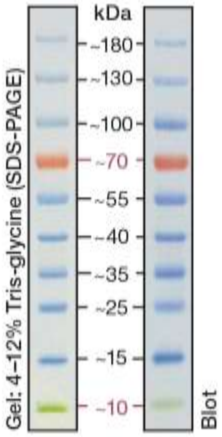

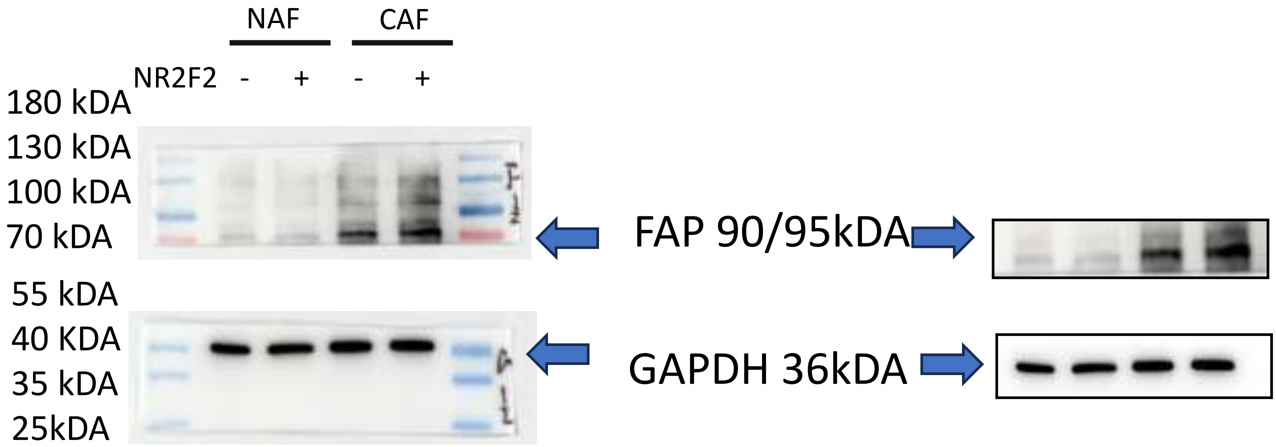

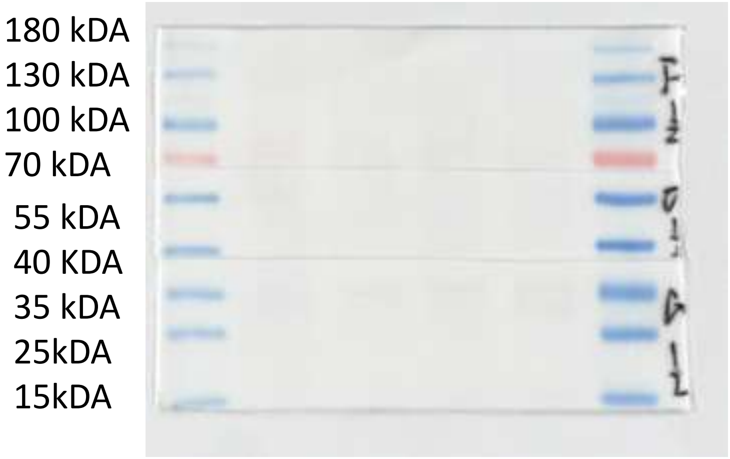

Full unedited blot for Figure 10A

marker 26616

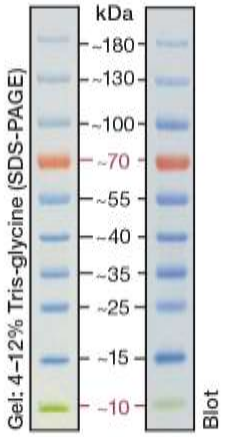

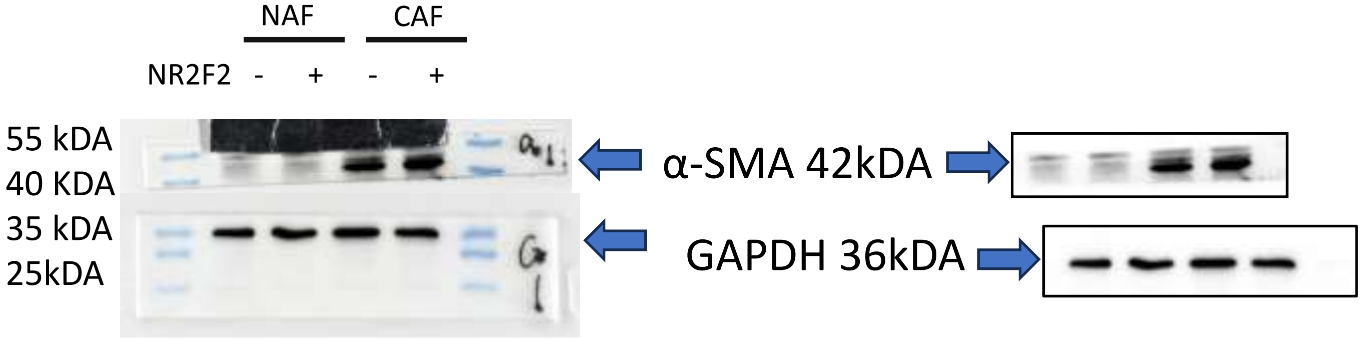

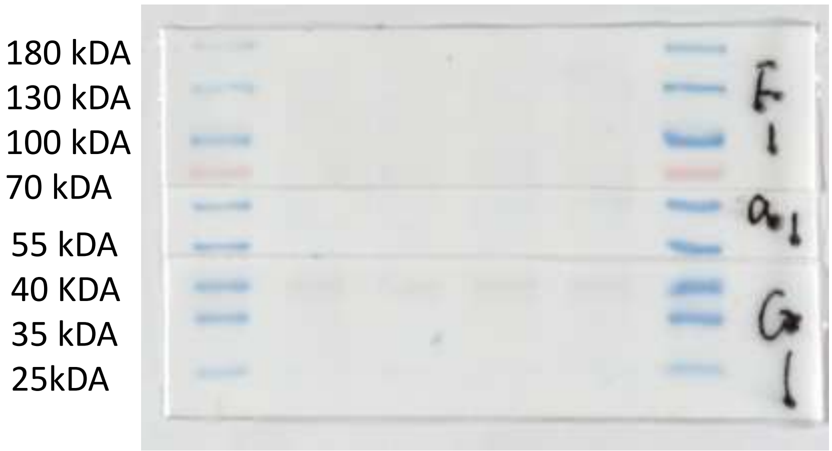

Full unedited blot for Figure 10A

marker 26616

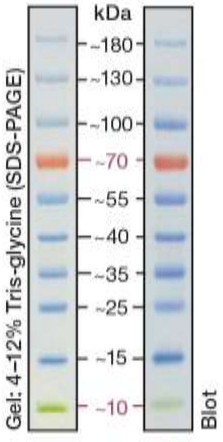

NAF CAF

NR2F2 - + - +

180 kDA
130 kDA
100 kDA
70 kDA

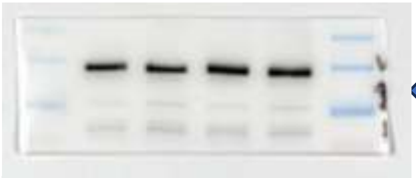

Vinculin 125kDA

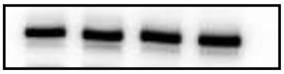

55 kDA
40 KDA
35 kDA
25kDA

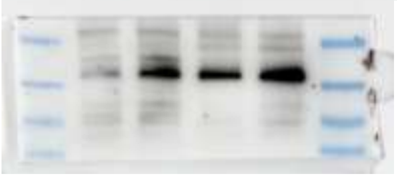

NR2F2 47kDA

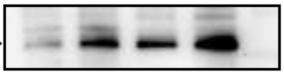

180 kDA
130 kDA
100 kDA
70 kDA
55 kDA
40 KDA
35 kDA
25kDA

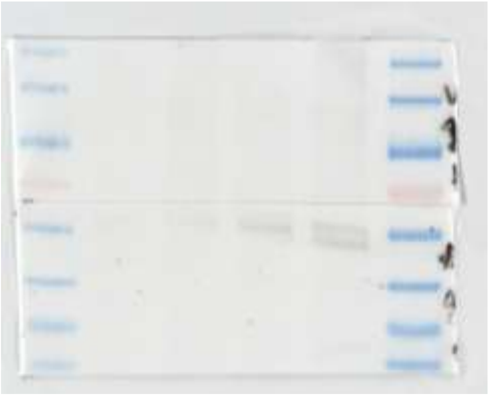

Full unedited blot for Figure S4 B

marker 26616

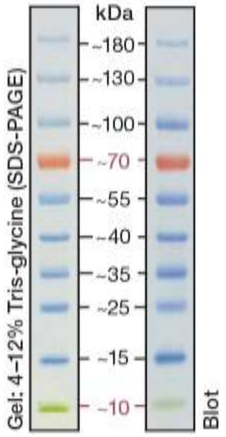

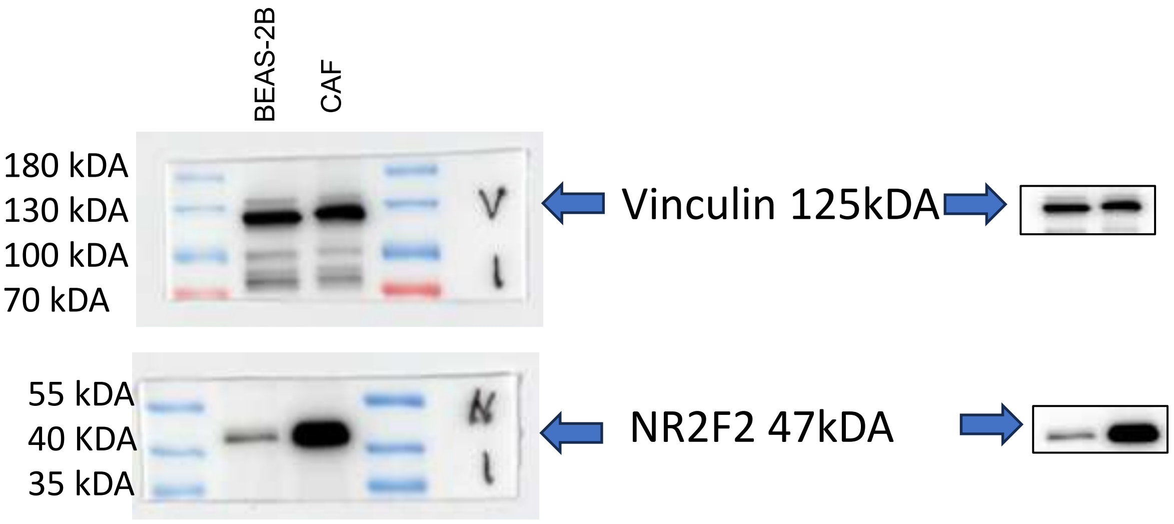

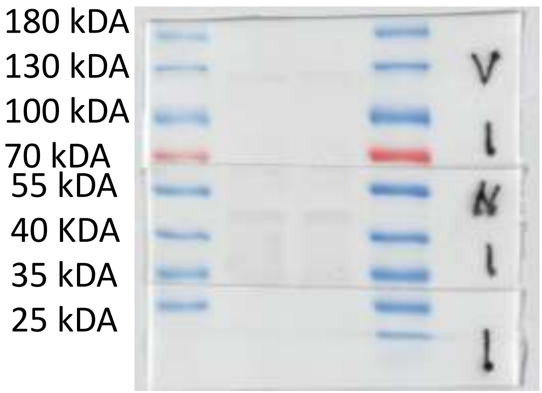

Full unedited blot for S4 C

marker 26616

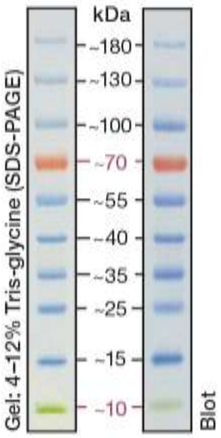

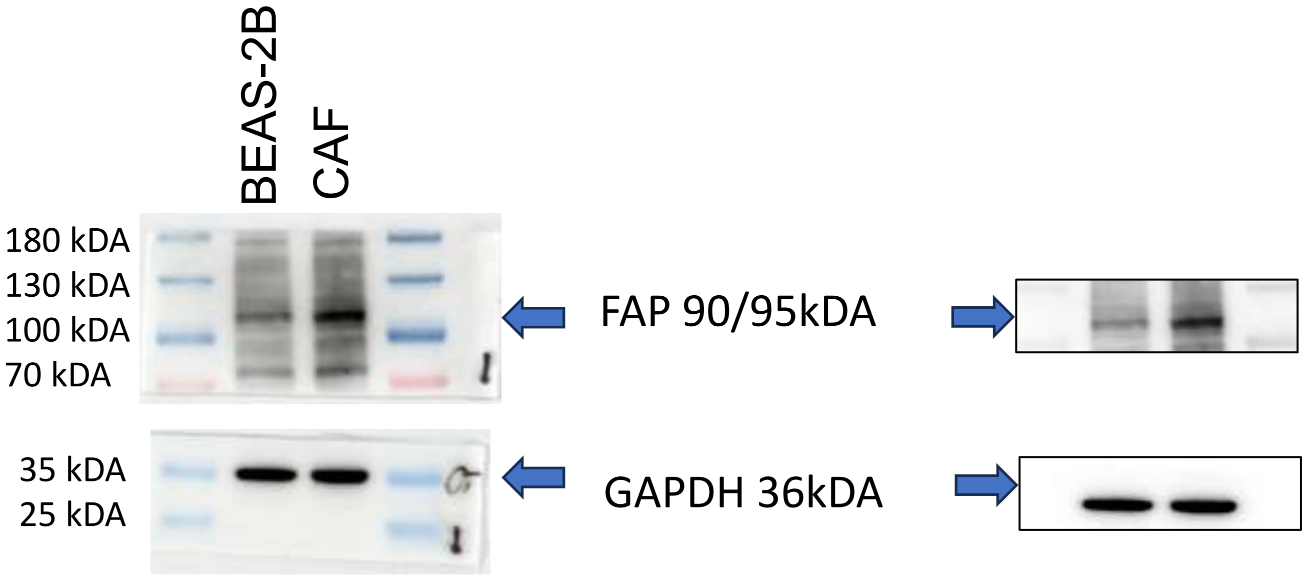

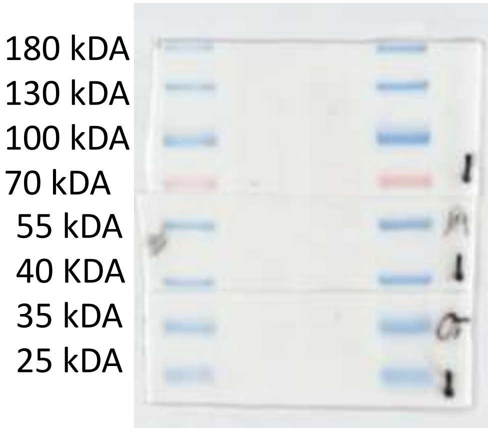

Full unedited blot for S4 C

marker 26616

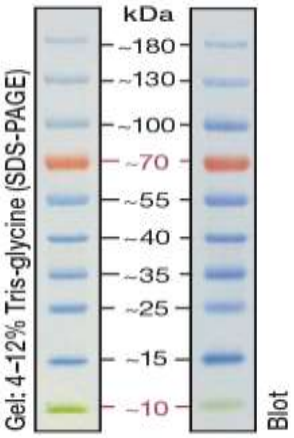

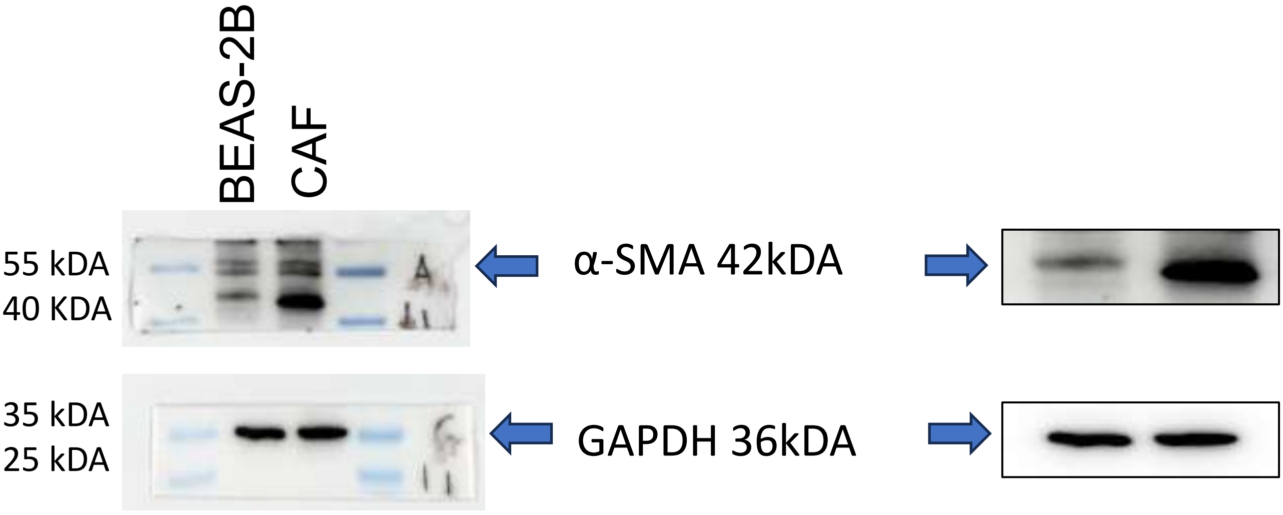

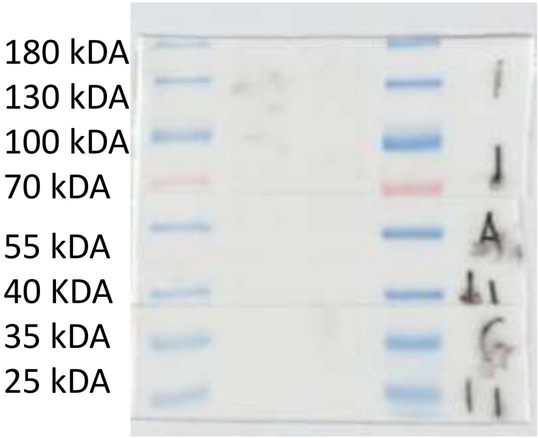

Full unedited blot for Figure S5 C

marker 26616

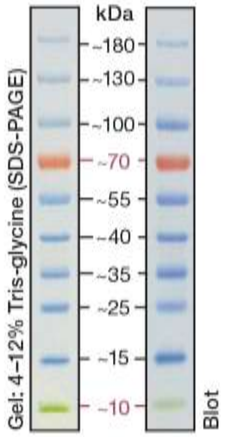

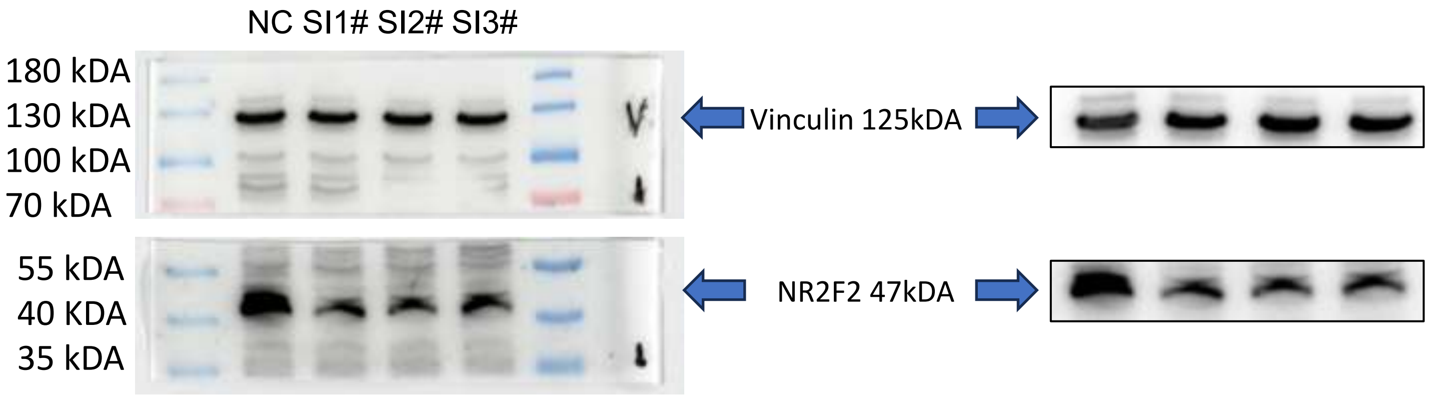

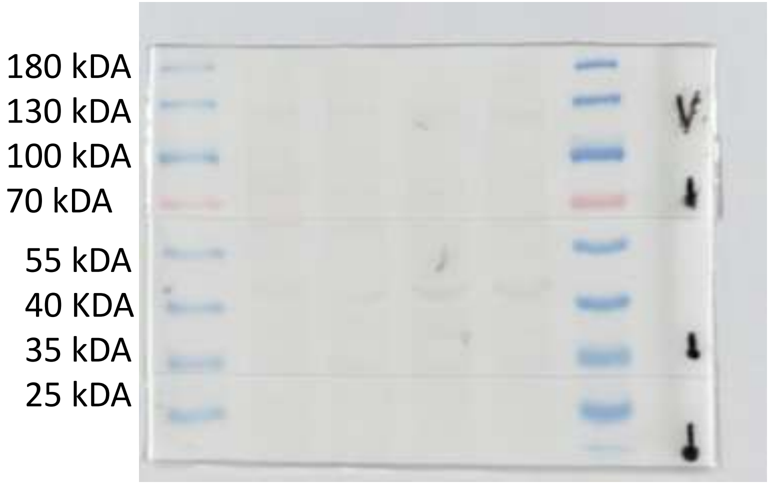

Supplement: Supplementary file 1 [file DataSheet1.pdf]
