# Supplementary figures and images for: NR2F2 in cancer-associated fibroblasts drives immune microenvironment remodeling and promotes lung adenocarcinoma progression
Source: Front Immunol. 2026 Apr 13;17:1776008. doi: 10.3389/fimmu.2026.1776008 (PMC13110960; doi:10.3389/fimmu.2026.1776008)

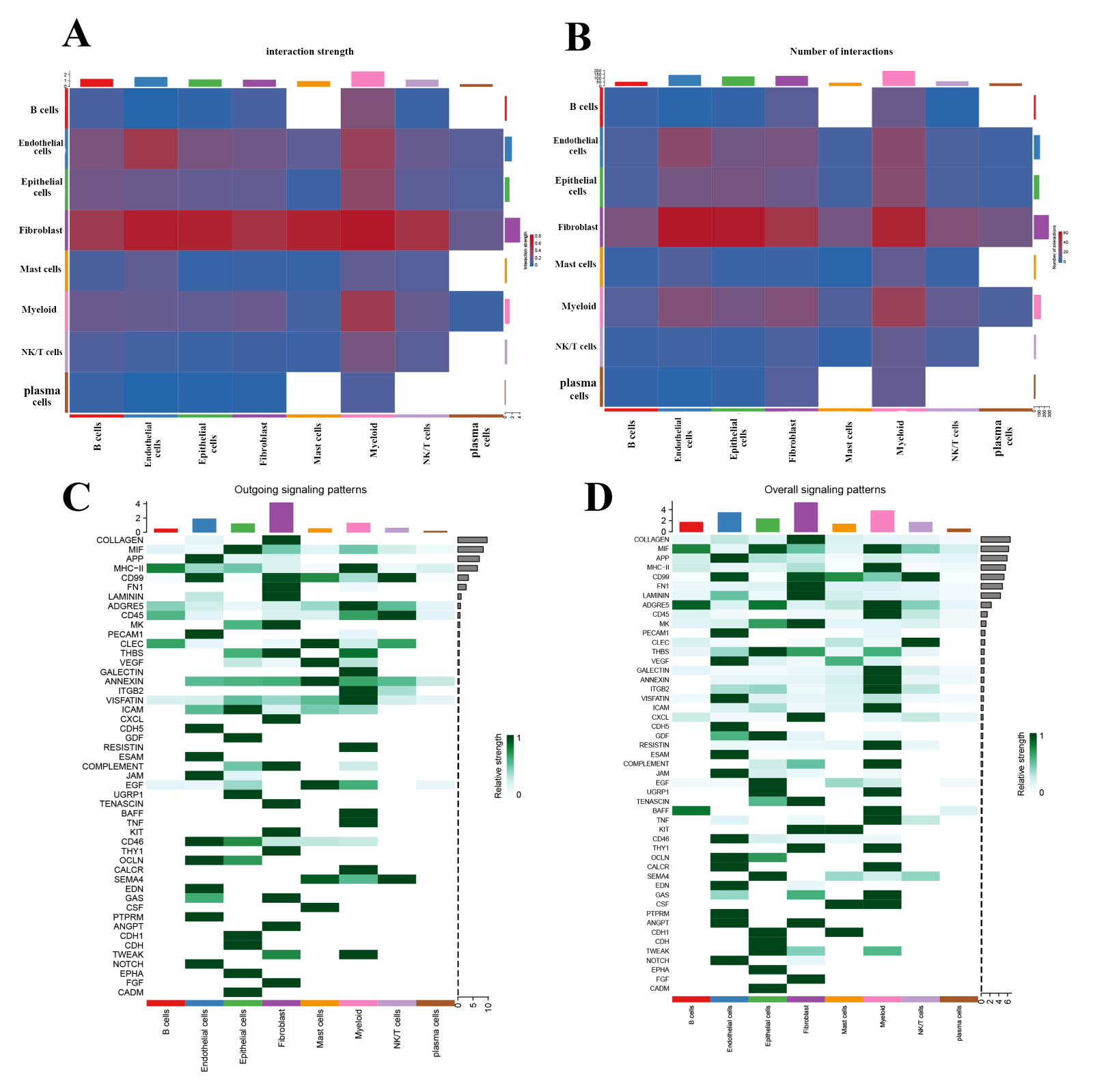

Supplement: Supplementary Figure 1 — Interaction strength (A) and number (B) among different cells. Heatmap of outgoing signaling patterns showing cell sending signal strength (left)(C)and total signaling strength (right) (D). [file Image1.tif]

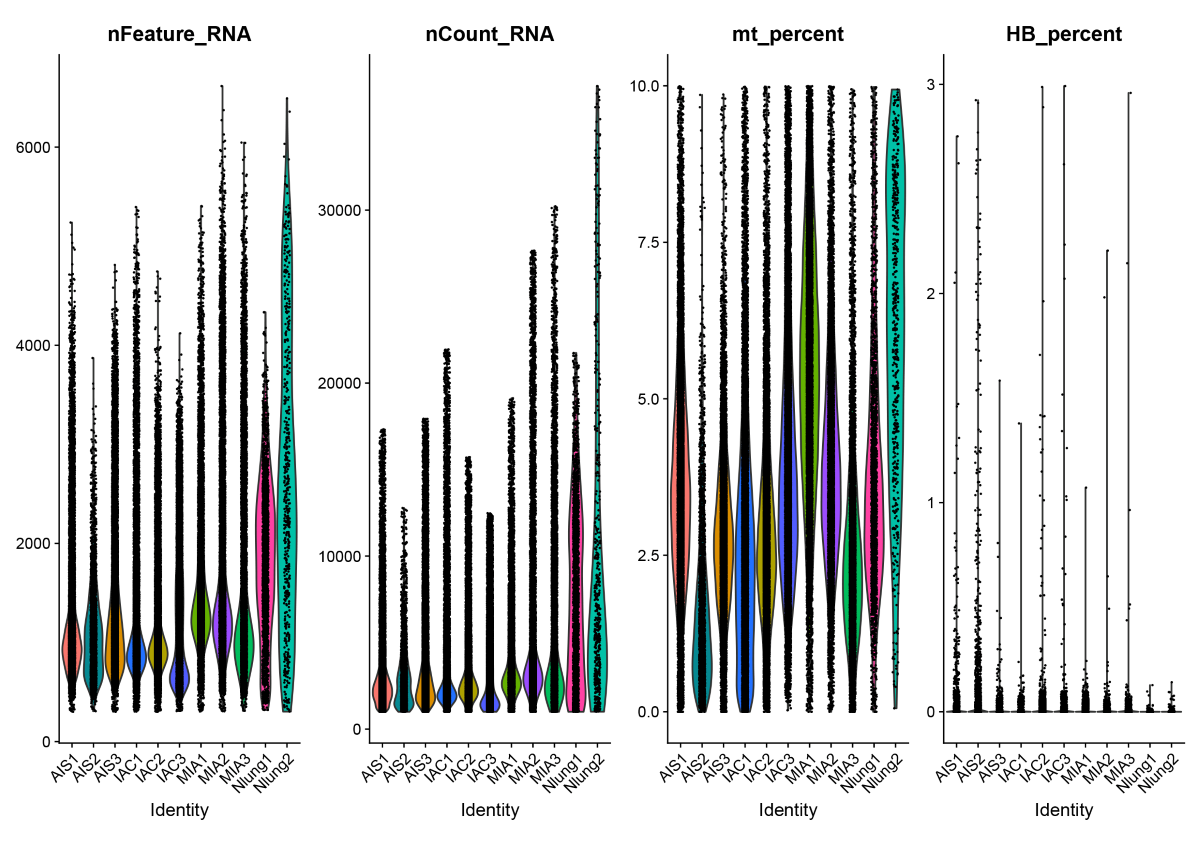

Supplement: Supplementary Figure 2 — Quality control of scRNA-seq data. [file Image2.tif]

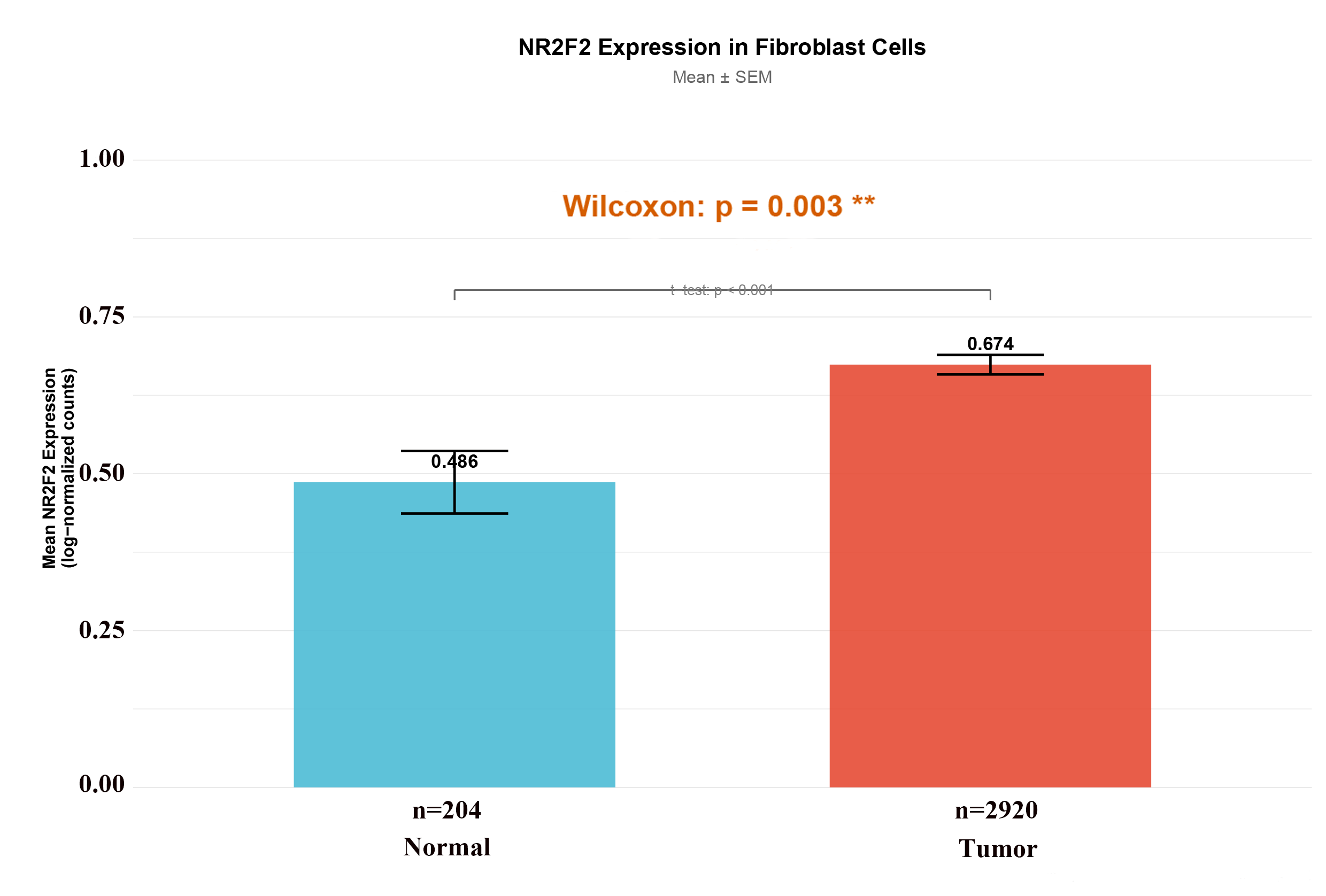

Supplement: Supplementary Figure 3 — Expression of NR2F2 in fibroblasts (scRNA-seq). [file Image3.tif]

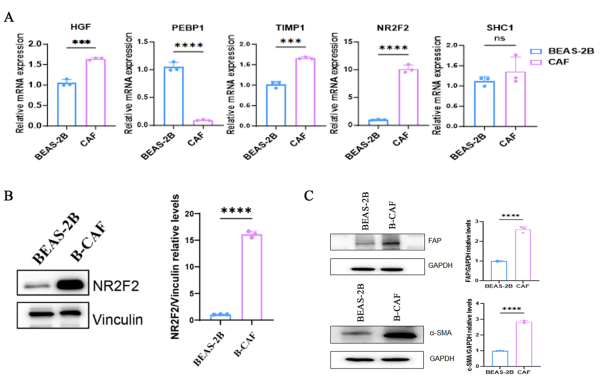

Supplement: Supplementary Figure 4 — (A) Expression of 5 candidate genes in BEAS-2B and B-CAFs (B) WB display of NR2F2 expression levels in BEAS-2B and B-CAFs. (C) Expression levels of CAF activation markers induced by BEAS-2B.n ≥ 3, *p < 0.05, **p < 0.01, ***p < 0.001, **** p < 0.0001. [file Image4.tif]

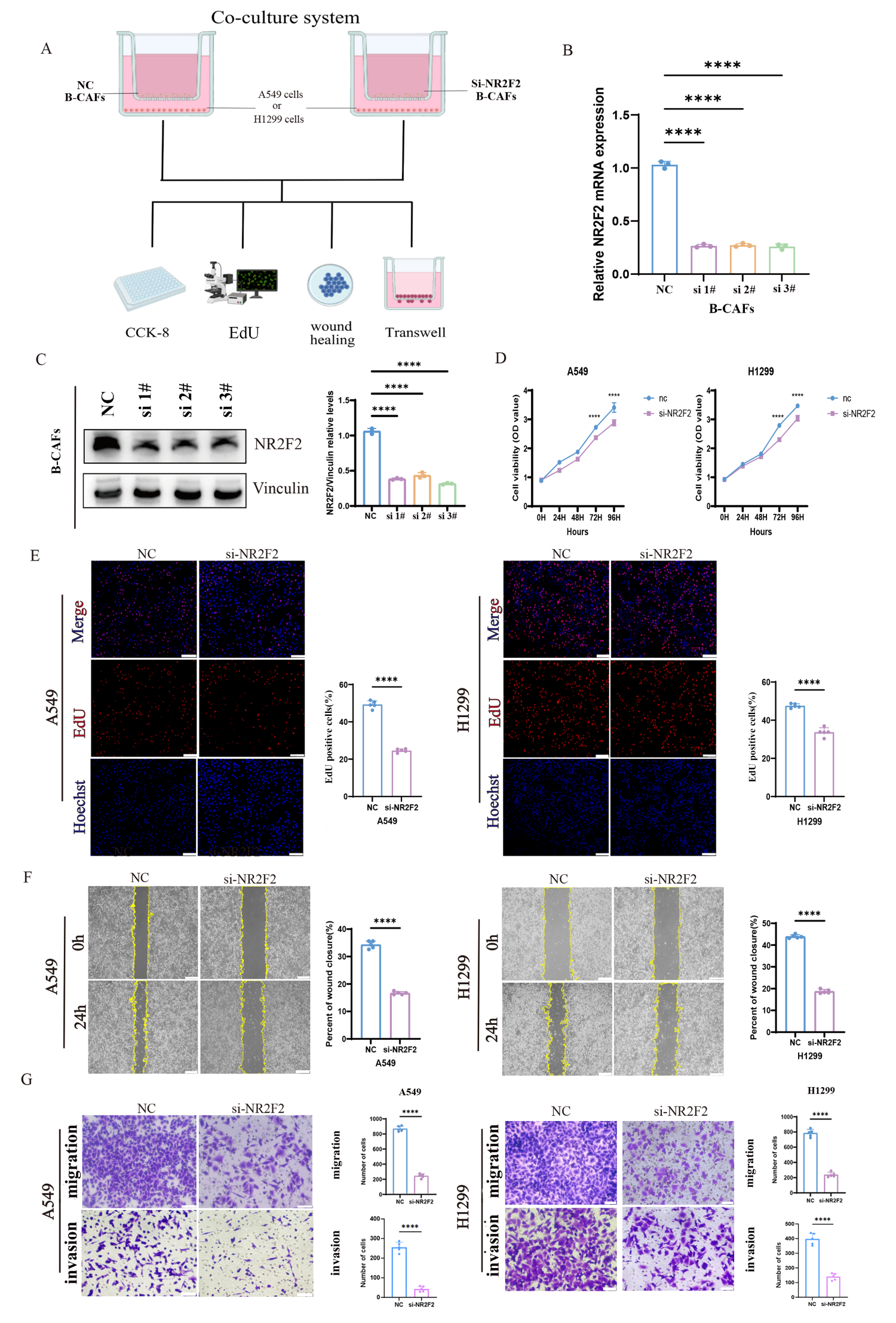

Supplement: Supplementary Figure 5 — Co-culture with NR2F2 knockdown CAFs reduces the proliferation, migration and invasion ability of tumor cells. (A) Schematic diagram of co-culture system of CAFs and tumor cells. (B) Efficiency validation of NR2F2 knockdown using RT-qPCR. (C) Efficiency validation of NR2F2 knockdown using Western Blot. (D) CCK8 experiment verifies proliferation efficiency. (E) EdU experiment verifies proliferation efficiency. (scale bar:200 µm) (F) Wound healing assessment verifies proliferation efficiency. (scale bar:400 µm) (G) Transwell assay verifies migration and invasion ability. (scale bar:200 µm) n ≥ 3, *p < 0.05, **p < 0.01, ***p < 0.001, **** p < 0.0001. [file Image5.tif]

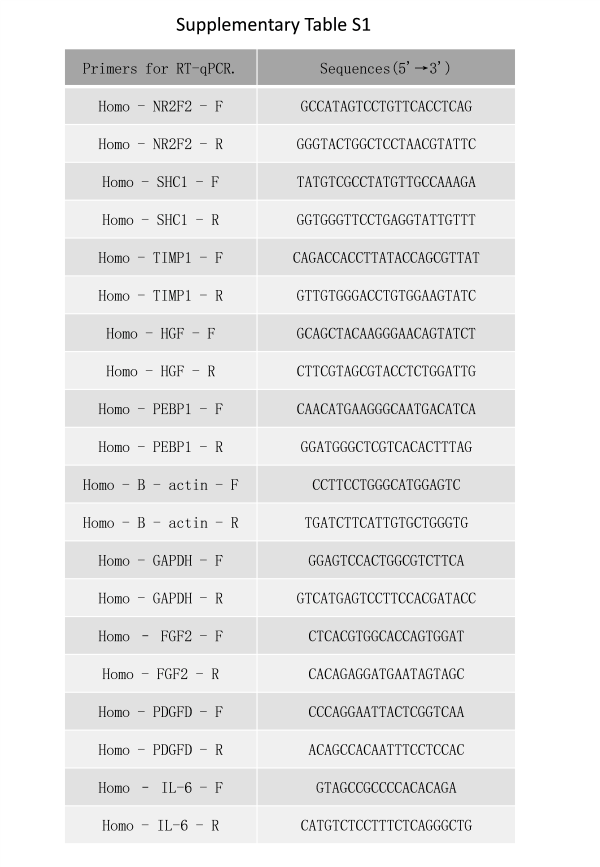

Supplement: Supplementary Table 1 — Primer sequences for RT-qPCR experiments. [file Image6.tif]

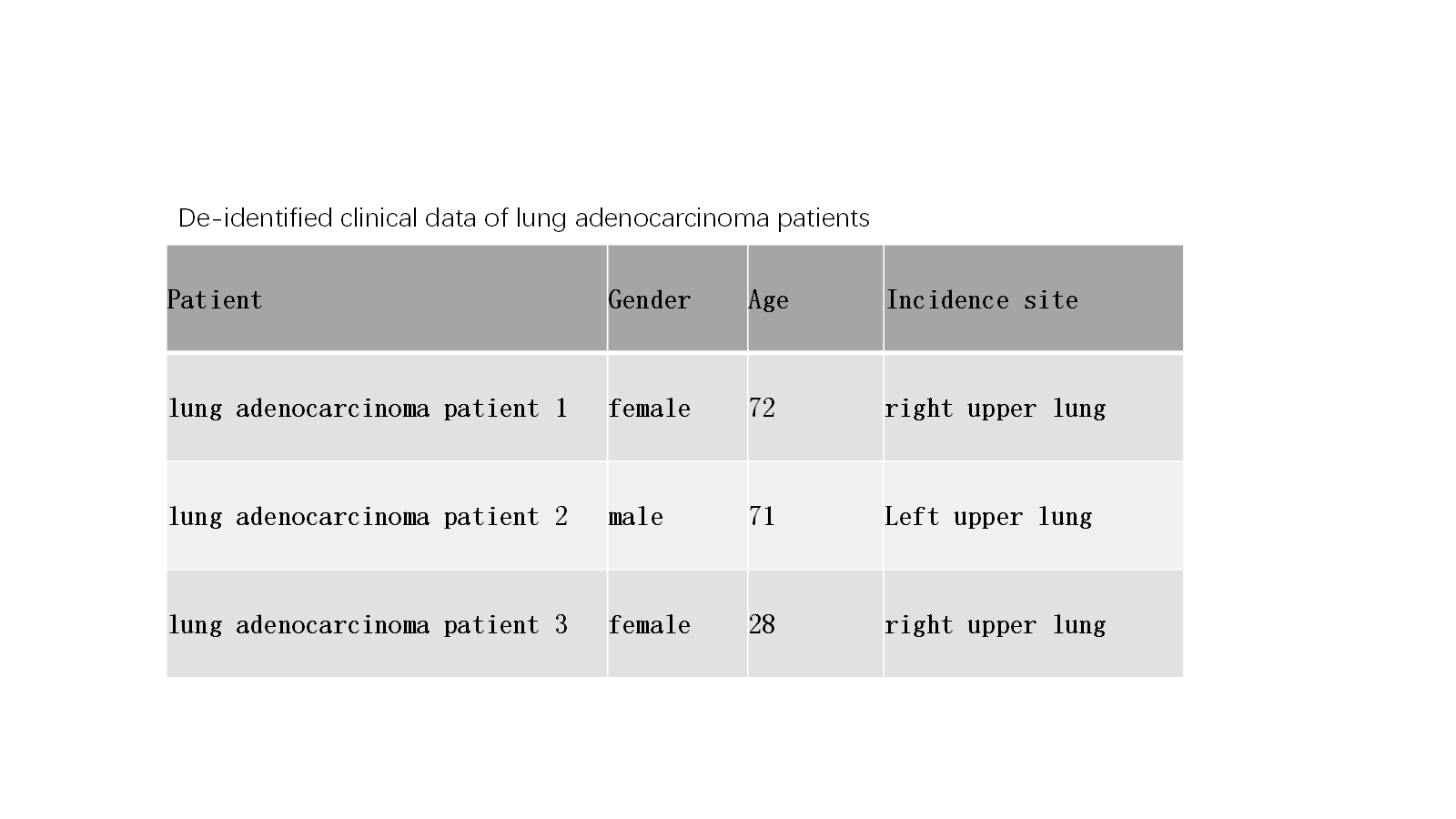

Supplement: Supplementary Table 2 — De-identified clinical data of lung adenocarcinoma patients. [file Image7.png]

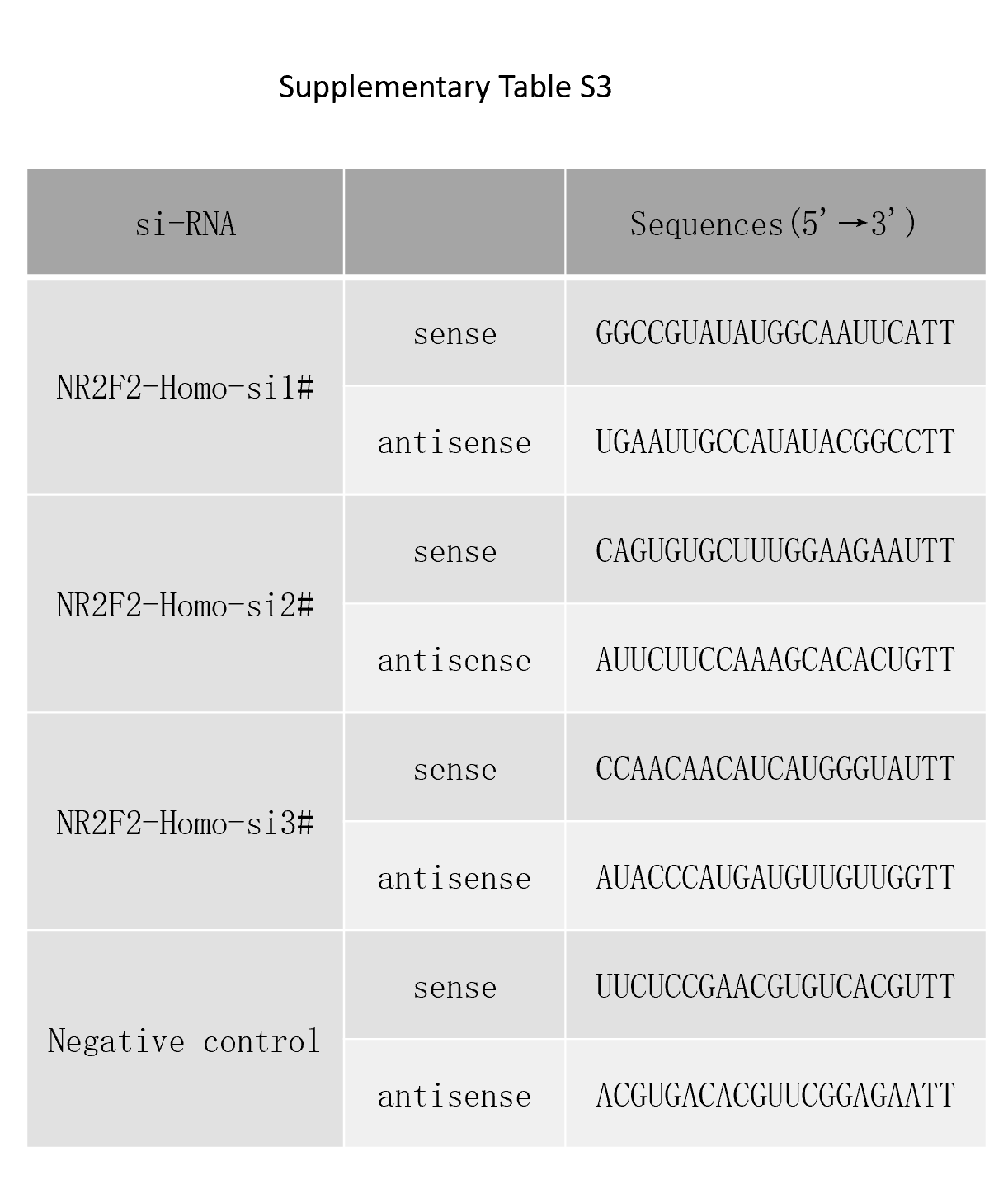

Supplement: Supplementary Table 3 — Si-RNA sequences. [file Image8.png]
